# The Impact of Various Poly(vinylpyrrolidone) Polymers on the Crystallization Process of Metronidazole

**DOI:** 10.3390/pharmaceutics16010136

**Published:** 2024-01-19

**Authors:** Luiza Orszulak, Taoufik Lamrani, Magdalena Tarnacka, Barbara Hachuła, Karolina Jurkiewicz, Patryk Zioła, Anna Mrozek-Wilczkiewicz, Ewa Kamińska, Kamil Kamiński

**Affiliations:** 1 Institute of Chemistry, Faculty of Science and Technology, University of Silesia in Katowice, Szkolna 9, 40-007 Katowice, Poland; barbara.hachula@us.edu.pl; 2 Institute of Physics, Faculty of Science and Technology, University of Silesia in Katowice, 75 Pułku Piechoty 1A, 41-500 Chorzow, Poland; taoufik.lamrani@us.edu.pl (T.L.); magdalena.tarnacka@us.edu.pl (M.T.); karolina.jurkiewicz@us.edu.pl (K.J.); patryk.ziola@us.edu.pl (P.Z.); anna.mrozek-wilczkiewicz@us.edu.pl (A.M.-W.); kamil.kaminski@us.edu.pl (K.K.); 3 Biotechnology Centre, Silesian University of Technology, Boleslawa Krzywoustego 8, 44-100 Gliwice, Poland; 4 Department of Pharmacognosy and Phytochemistry, Faculty of Pharmaceutical Sciences in Sosnowiec, Medical University of Silesia in Katowice, Jagiellonska 4, 41-200 Sosnowiec, Poland; ekaminska@sum.edu.pl

**Keywords:** metronidazole, polyvinylpyrrolidone, topology, star-shaped polymer, amorphous solid dispersion, binary mixtures

## Abstract

In this paper, we propose one-step synthetic strategies for obtaining well-defined linear and star-shaped polyvinylpyrrolidone (*lin*PVP and *star*PVP). The produced macromolecules and a commercial PVP K30 with linear topology were investigated as potential matrices for suppressing metronidazole (MTZ) crystallization. Interestingly, during the formation of binary mixtures (BMs) containing different polymers and MTZ, we found that linear PVPs exhibit maximum miscibility with the drug at a 50:50 weight ratio (*w*/*w*), while the star-shaped polymer mixes with MTZ even at a 30:70 *w*/*w*. To explain these observations, comprehensive studies of MTZ-PVP formulations with various contents of both components were performed using Fourier-transform infrared spectroscopy, differential scanning calorimetry, and X-ray diffraction. The obtained results clearly showed that the polymer’s topology plays a significant role in the type of interactions occurring between the matrix and MTZ. Additionally, we established that for MTZ-PVP 50:50 and 75:25 *w*/*w* BMs, linear polymers have the most substantial impact on inhibiting the crystallization of API. The star-shaped macromolecule turned out to be the least effective in stabilizing amorphous MTZ at these polymer concentrations. Nevertheless, long-term structural investigations of the MTZ-*star*PVP 30:70 *w*/*w* system (which is not achievable for linear PVPs) demonstrated its complete amorphousness for over one month.

## 1. Introduction

For several decades, the pharmaceutical sector has faced a significant challenge in improving the solubility and bioavailability of traditional, i.e., crystalline, drugs, aiming to enhance the effective delivery of medications. Unfortunately, a large percentage of drugs available on the market (approximately 40%) and those in the research and development phase (approximately 90%) still exhibit poor water solubility, which significantly hampers their absorption and consequently leads to less effectiveness in the human body [1,2,3,4]. Therefore, in recent years, the use of amorphous active pharmaceutical ingredients (APIs) is gaining increasing interest, as they often provide better solubility, dissolution rate, and bioavailability compared to their crystalline counterparts. On the other hand, due to high free energy, APIs in this form reveal a high risk of recrystallization over time, leading to a potential loss of therapeutic efficacy [5,6,7,8]. Hence, improving physical stability during processing and storage is crucial for the development of effective pharmaceuticals. A commonly employed method for stabilizing amorphous APIs is the formation of binary mixtures (BMs) with polymeric excipients (EXCs), referred to as amorphous solid dispersions (ASDs) [9]. In the literature, several mechanisms have been proposed to interpret the impact of macromolecules on the stability of drugs in ASDs [10,11]. For example, it has been demonstrated that hydrogen bonds formed between polymeric matrices and APIs enhance the physical stability of amorphous pharmaceuticals by reducing molecular mobility [12,13]. There are also reports showing that other types of interactions, such as ionic, dipole–dipole, and van der Waals interactions, inhibit the recrystallization of APIs from the amorphous binary systems [14,15]. Moreover, one can mention the volume fraction effect [16,17,18,19], as well as the increase in the glass transition temperature (T_g_) and consequently, the reduced molecular mobility of the system, due to the addition of the polymer [12,20]. The crucial role of high-molecular-weight systems prompts scientists to conduct further in-depth studies on the ASDs of active substances with various kinds of polymers.

Herein, it should be pointed out that polymeric EXCs used in ASDs are subject to high requirements. These compounds must exhibit non-toxicity, compatibility with human tissues, and have a well-defined structure [21]. The most commonly applied macromolecules in amorphous BMs belong to three classes [22]: (i) cellulose derivatives (e.g., hydroxypropyl methylcellulose, hydroxypropyl methylcellulose acetate succinate, hydroxypropyl methylcellulose phthalate) [23,24,25,26], (ii) polyvinylpyrrolidone (PVP) and its copolymers (e.g., polyvinylpyrrolidone/vinyl acetate copolymer, PVP/VA) [27,28,29,30,31], and (iii) methacrylic acid and methacrylate esters (e.g., Eudragit^®^ L100, S100) [32,33,34]. Undoubtedly, among the listed polymers widely used in the pharmaceutical industry, PVP stands out for its particularly advantageous properties. This macromolecule is non-toxic, inert, temperature-resistant, pH-stable, and biocompatible [21]. Moreover, it shows a complex affinity for hydrophilic and hydrophobic drugs, which is especially desirable in the formulation of BMs [35,36,37,38]. As shown in the literature, the majority of studies primarily focus on the impact of commercially available PVP on the crystallization of APIs in ASDs [39,40,41,42]. For example, Balani et al. reported that the addition of 33% of PVP K30 significantly suppresses the recrystallization of amorphous salbutamol sulfate, while an 80% polymer content completely stabilizes the amorphous form of the drug (even after a storage period) [39]. Herein, it should be noted that the large-scale production of PVP occurs through uncontrolled radical polymerization, allowing only for the attainment of poorly defined macromolecules (a broad range of molecular weights M_n_ and hence, high dispersity Ð) [43,44]. Meanwhile, the most desirable polymers in the pharmaceutical industry are macromolecules with precisely tailored parameters, characterized by the target M_n_, low-to-moderate Ð (preferably not exceeding a value of 1.5), and possessing active chain ends enabling further modifications according to specific requirements. Unfortunately, researchers in numerous papers have still only considered the influence of molecular weight and concentration of PVP (e.g., Kollidon^®^ K10, K30, K90) on the physical stability of drug–polymer blends [28,42,45,46]. On the other hand, aside from one paper devoted to PVP mixed with flutamide [37], the impact of microstructure/tacticity, as well as the dispersity and topology of this polymer, on the physico-chemical and pharmacokinetic properties of APIs is almost completely overlooked, creating an exceptionally intriguing research gap.

Hence, it seems relevant to perform more systematic studies to verify whether the microstructure, dispersity, or importantly topology of the polymer affect the crystallization kinetics and physical stability of a given pharmaceutical. In this context, metronidazole (MTZ), the antibacterial and antiprotozoal medication [47,48], is looming as an ideal candidate for such investigations. Briefly, one can stress that the amorphization of this active substance is a difficult task. For example, it is not possible to obtain it in the glassy form using the standard vitrification method due to its immediate crystallization after melting [49]. So far, only one successful attempt to produce a stable amorphous MTZ in ASDs has been reported. Minecka et al. have demonstrated that acetylated cyclodextrins (especially the β form) can effectively inhibit the recrystallization of MTZ from the glassy state [49]. Therefore, further exploration of novel EXCs (both low- and high-molecular-weight) effectively stabilizing amorphous MTZ appears fully justified.

In this paper, we examine BMs of MTZ with three polymers, i.e., (i) commercial linear PVP (with high Ð), (ii) self-synthesized well-defined linear PVP (with low Ð), and (iii) self-synthesized well-defined star-shaped PVP (with low/moderate Ð). Herein, one can add that the latter two macromolecules are not available on the market. The main aim of our studies, carried out using differential scanning calorimetry (DSC), X-ray diffraction (XRD), as well as infrared spectroscopy (FTIR) techniques, is to explain how the addition of PVP with different topologies and well-defined structures (M_n_, Ð) influences the recrystallization tendency of MTZ. Understanding the impact of polymer structure on the crystallization rate of APIs is of paramount importance in the rational design of amorphous formulations with excellent physical stability.

## 2. Materials and Methods

### 2.1. Materials

1-vinyl-2-pyrrolidone (VP, >99%, Sigma Aldrich, Poznan, Poland) was passed through an alumina column before use to remove the inhibitor. 2,2′-azobis(2-methylpropionitrile) solution (AIBN, 0.2 M in toluene, Sigma Aldrich), cyanomethyl methyl(4-pyridyl) carbamodithioate (CTA1, 98%, Sigma Aldrich), 1,3,5-tris(bromomethyl)benzene (97%, Sigma Aldrich), sodium diethyldithiocarbamate trihydrate (Sigma Aldrich), diethyl ether (pure for analysis, Chempur, Piekary Slaskie, Poland), methanol (99.85%, PureLand, Stargard, Poland), dichloromethane (DCM, 99%,, Karpinex, Warszawa, Poland), chloroform-d (99.8% D, contains 0.03% *v*/*v* TMS, Sigma Aldrich), PVP K30 (M_n_ = 40,000 g/mol, Ð = 2.05, Sigma Aldrich), and crystalline MTZ (IUPAC name 2-(2-Methyl-5-nitro-1H-imidazol-1-yl)ethanol, 99%, Sigma Aldrich) were used as received.

### 2.2. The Procedure of Preparing Amorphous MTZ-PVP BMs

Amorphous binary mixtures composed of MTZ and PVP polymers, including a commercial PVP K30, and synthesized linear and star-shaped PVP samples were obtained using the melt-cooling method. The MTZ-PVP systems were prepared at different weight ratios of API to polymer, i.e., 75:25, 60:40, 50:50. In the case of MTZ-starPVP, it was also possible to prepare 40:60 and 30:70 *w*/*w* systems. To obtain a homogeneous mixture, appropriate amounts of crystalline MTZ and PVP were weighed, carefully transferred to a metal plate, and preliminarily mixed using a spatula. Subsequently, the plate with the API–polymer mixture was transferred to a hot plate heated to a temperature of 438 K. After a while, MTZ began to melt and the entire BM was stirred until the complete dissolution of PVP in the API. After determining a homogeneous system, the sample was vitrified by rapidly transferring it to a pre-cooled copper plate.

### 2.3. Nuclear Magnetic Resonance (NMR)

^1^H and ^13^C NMR spectra for the samples dissolved in chloroform-d (with tetramethylsilane internal standard) were collected using a Bruker Ascend 600 MHz spectrometer. For measurements, standard experimental conditions and the standard Bruker program were applied.

### 2.4. Size Exclusion Chromatography (SEC)

M_n_ and Ð of linear and star-shaped PVP homopolymers were determined by size exclusion chromatography (SEC). For data collection, we used a Viscotek GPC Max VE 2001 (Malvern, UK) and a Viscotek TDA 305 (Malvern, UK) triple detection system (refractometer, viscosimeter, and low-angle laser light scattering). The OmniSec 5.12 was applied for data processing. The separation was carried out using two T6000M general mixed columns. The measurements were performed in DMF/LiBr (0.01 M) as an eluent at T = 303 K, with a flow rate of 0.7 mL/min.

### 2.5. Cell Culture

Normal human dermal fibroblasts (NHDFs) were purchased from PromoCell. The cell line was cultured in Dulbecco’s Modified Eagle’s Medium (DMEM) supplemented with 15% non-inactivated FBS (Sigma Aldrich) and penicillin/streptomycin antibiotics (1% *v*/*v*; Gibco, Waltham, MA, USA). The cells were kept under standard conditions at 37 °C in a humid atmosphere with 5% CO_2_ and passed as required by the manufacturer. Additionally, cells were tested against Mycoplasma contamination using the PCR technique with specific primers.

### 2.6. Cytotoxicity Studies

The cells were seeded in 96-well transparent plates (Nunc, Waltham, MA, USA) at a density of 4000 cells per well and incubated for 24 h at 37 °C. Approximately 24 h after seeding, the medium was removed from wells and 200 µL of various concentrations (from 0.05 to 0.001 mg/mL) of tested compounds (dissolved in DMEM) were added to the plate and incubated for 72 h at 37 °C. Next, compound solutions were exchanged with 100 µL of DMEM without phenol red and 20 µL of CellTiter 96^®^AQueous One Solution-MTS (Promega, Madison, WI, USA) and incubated for one hour at 37 °C. After that time, the absorbance of the samples was measured at 490 nm using a multi-plate reader (Varioskan LUX, Thermo Scientific, Waltham, MA, USA). The results were normalized to a control consisting of untreated cells. Compounds were tested in triplicate in a single experiment, each repeated three times.

### 2.7. Fourier-Transform Infrared (FTIR) Spectroscopy

FTIR spectra were collected on a Nicolet iS50 FTIR spectrometer (Thermo Scientific) with a built-in diamond iS50 ATR sampling station in the MIR range of 4000–400 cm^−1^. Each spectrum was recorded from 16 scans with a resolution of 4 cm^−1^. The high-temperature FTIR spectrum of MTZ (at the melting point of 438 K) was measured using a GladiATR accessory (Pike Technologies, Madison, WI, USA) coupled with an FTIR spectrometer in the range 4000–400 cm^−1^ (16 scans; spectral resolution of 2 cm^−1^).

### 2.8. Differential Scanning Calorimetry (DSC)

Calorimetric measurements of MTZ, various PVPs (i.e., PVP K30, *lin*-PVP, and *star*-PVP), and their BMs were performed using a Mettler-Toledo DSC system, which is equipped with a liquid nitrogen cooling accessory and an HSS8 ceramic sensor. Temperature and enthalpy were calibrated using indium and zinc standards. Samples were placed in aluminum crucibles (40 µL). DSC measurements were carried out in a temperature range of 250 to 480 K. The heating/cooling rate (*ϕ*) during experiments was equal to 10 K/min.

Additionally, for all studied MTZ-PVP BMs, non-isothermal DSC measurements were performed over a temperature range of 250 to 460 K. They were made at a *ϕ* from 2.5 to 15 K/min.

### 2.9. X-ray Diffraction (XRD)

X-ray diffraction measurements were conducted employing a D/Max Rapid II diffractometer (Rigaku Corporation, Akishima-shi, Japan) equipped with an image plate detector, a rotating silver anode, and an incident beam (002) graphite monochromator. The wavelength (*λ*) of the incident beam was 0.56 Å. Samples were measured in borosilicate glass capillaries in the Debye–Scherrer geometry. The diffraction patterns were collected as functions of the scattering angle (2θ) in a single shot using a 2D curved image plate detector and transformed to functions of the scattering vector Q=4π sin(θ)/λ. Samples containing recrystallized MTZ were subjected to measurements over 1 h. The measurement time for freshly prepared ASDs was limited to 15 min due to their high recrystallization rate. The temperature during all measurements was 295 K.

## 3. Results and Discussion

In the first stage of our studies, we developed procedures for obtaining well-defined linear (*lin*PVP) and three-arm star-shaped PVP (*star*PVP) homopolymers, which are thermodynamically stable amorphous macromolecules. Herein, it should be noted that despite PVP being a widely used polymer in the pharmaceutical and cosmetic industries [50], only linear PVP (with various molecular weights) and its copolymers are currently available in the commercial market. In contrast, other topologies of this polymer are entirely unavailable on a large scale. Therefore, the search for synthetic methods enabling the production of well-defined non-linear PVP polymers is an extremely interesting and ambitious research topic. A promising technique for producing complex macromolecular structures appears to be Reversible Addition–Fragmentation Chain Transfer (RAFT) polymerization, where a crucial role is played by a suitably selected Chain Transfer Agent (CTA) for a specific type of monomer [51]. As indicated in the literature, suitable CTAs for *N*-vinyl monomers (including VP) are compounds from the xanthate and dithiocarbamate groups [52,53]. It should also be emphasized that a significant majority of commercially available CTAs are single-functional compounds, allowing for the formation of linear macromolecules. In fact, one paper describes the successful synthesis of a four-arm star-shaped PVP using the RAFT method [54]. However, it is worth noting that the article’s authors utilized a commercially available tetrafunctional CTA from the trithiocarbonate group, which is not considered to control the polymerization of *N*-vinyl monomers [52,53]. As a consequence, attempts to conduct RAFT polymerization at ambient pressure and using the discussed CTA led to the production of star polymers with high dispersities, Ð (up to 2.45). Interestingly, RAFT polymerization at higher pressure (*p* = 250 MPa) yields a four-arm star-shaped PVP with a moderate Ð = 1.80.

**Figure 1:**
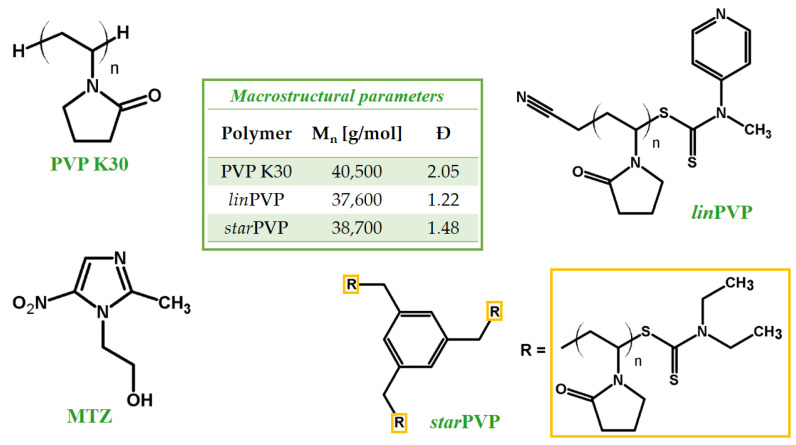
The chemical structures of metronidazole (MTZ) and the employed PVP polymers (K30, *lin*PVP, *star*PVP) along with a table presenting their macrostructural parameters.

Based on the information presented above and noting the lack of commercially available multifunctional CTAs tailored for *N*-vinyl monomers, we decided to independently obtain a trifunctional CTA from the dithiocarbamate group. Subsequently, it was used to synthesize a well-defined three-arm star-shaped PVP (M_n_ =38,700 g/mol; Ð = 1.48) using a *“core-first”* strategy. In turn, a commercial CTA also from the dithiocarbamate group was applied to synthesize linear PVP, resulting in a homopolymer with favorable macrostructural parameters (M_n_ =37,600 g/mol; Ð = 1.22) (see Figure 1). The description of the synthesis of the trifunctional CTA and PVP homopolymers (*lin*PVP and *star*PVP) is included in the Supplementary Materials (SMs).

### 3.1. Cytotoxicity Studies

**Figure 2:**
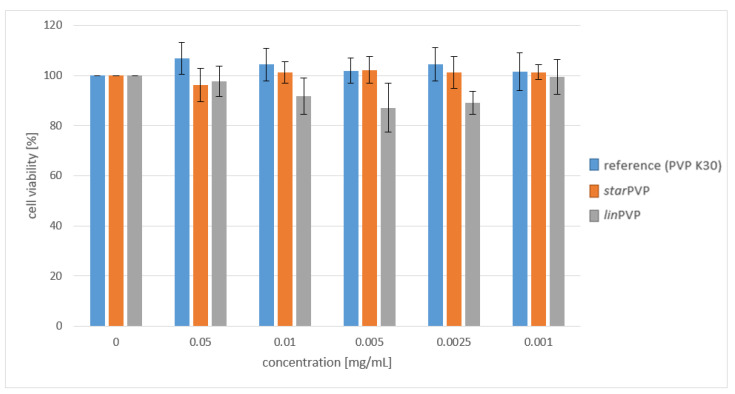
Cell viability seeded onto various PVP samples.

Since both synthesized *lin*PVP and *star*PVP are new materials, we decided to perform cytotoxicity tests on normal human dermal fibroblasts (NHDFs) first and compare the results with the data obtained for the commercially available PVP K30, which is thoroughly used in pharmaceutical formulations. Herein, it should be briefly mentioned that we carried out measurements with a PVP concentration no higher than 0.05 mg/mL as is usually conducted for macromolecules [55,56,57,58]. Figure 2 presents a graph of the dependence of cell survival on the concentration of the examined compounds. As illustrated, the tested polymers show no cytotoxicity against the normal human dermal fibroblast line. The survival fraction in all cases did not fall below 85%, which indicates the neutral effect of the samples on the proliferation of the tested cell line. The studies revealed that end groups with a CTA moiety in the structure of the synthesized polymer as well as the presence of a star initiator do not affect their cytotoxicity with respect to PVP K30. Such a result is extremely promising in the context of the potential use of these materials as excipients in binary formulations.

### 3.2. Preparation of Amorphous Binary Mixtures of MTZ with PVP

Having cytotoxicity data at hand, we proceeded to prepare amorphous BMs of three different PVPs with similar molecular weights (M_n_ ≈ 40,000 g/mol) but different dispersities and topologies with MTZ—a pharmaceutical with a high tendency to crystallize. Herein, it is worth pointing out that PVP K30 exhibits relatively high dispersity (Ð = 2.05) compared to the synthesized *lin*PVP and *star*PVP. It implies that this polymer consists of both short and long macromolecular chains, most likely due to an uncontrolled synthesis procedure on a large/industrial scale [59]. It should also be noted that each macromolecule was fully amorphous and thermodynamically stable.

Importantly, during the samples’ preparation, it was found that *star*PVP mixes much better with MTZ than the other two linear macromolecules (PVP K30, *lin*PVP). Consequently, it was possible to prepare homogeneous (verified further by DSC studies) mixtures up to 30 wt% of MTZ, while in other cases, 50 wt% was a limiting drug concentration. Such a result was quite surprising for us since we expected similar miscibility properties of various PVPs and MTZ. To understand this peculiar finding, FTIR investigations on all prepared samples were carried out.

### 3.3. Fourier-Transform Infrared Spectroscopy (FTIR) Data

To gain insight into the intermolecular interactions between given macromolecules and API, Attenuated Total Reflection (ATR)-FTIR measurements for the individual components (MTZ, PVPs) and their binary systems were performed at room temperature (T = 295 K) in the spectral range from 4000 to 400 cm^−1^. As it is impossible to obtain amorphous MTZ using the standard vitrification method due to its rapid crystallization after melting, we collected the FTIR spectrum of the molten MTZ to detect spectral differences between the crystalline and liquid samples. The spectra of the crystalline and molten MTZ, as well as three PVP samples, together with the assignment of characteristic bands to the appropriate molecular vibrations [49,60,61,62,63], are given in the SMs (see Figure S7), whereas the spectra of MTZ- PVP BMs in the supercooled liquid/or glassy and crystalline states, recorded in the regions of 3800–2400 and 1800–400 cm^−1^, are shown in Figures S8–S10. It is worth mentioning that in the crystal, MTZ molecules are linked through O—H···N hydrogen bonds and stabilized by C—H···O weak interactions, forming chains propagating/extending along the c-axis direction [64]. These interactions are reflected in the higher frequency range of the IR spectrum in the form of an intense and broad band located at 3204 cm^−1^ associated with the stretching vibration of the OH group, $\nu_{OH}$ (Figure S7). In the case of molten MTZ, the $\nu_{OH}$ band is shifted towards higher wavenumbers (blue shift, 3228 cm^−1^). The detailed assignment of other spectral bands of the MTZ sample and the peaks occurring in the spectra of three PVP samples is presented in the SMs. It is worth noting that the analyzed PVP spectra (PVP-K30, *lin*PVP, *star*PVP) essentially differ in the position of the $\nu_{OH}$ band originating from water adsorbed into the polymer (PVP-K30 $\nu_{OH}$ = 3409 cm^−1^, *lin*PVP $\nu_{OH}$ = 3436 cm^−1^, *star*PVP $\nu_{OH}$ = 3470 cm^−1^), and the position of the band related to the C=O stretching vibrations (PVP-K30 $\nu_{C=O}$ = 1645 cm^−1^, *lin*PVP $\nu_{C=O}$ = 1651 cm^−1^, *star*PVP $\nu_{C=O}$ = 1661 cm^−1^) (Figure S7).

**Figure 3:**
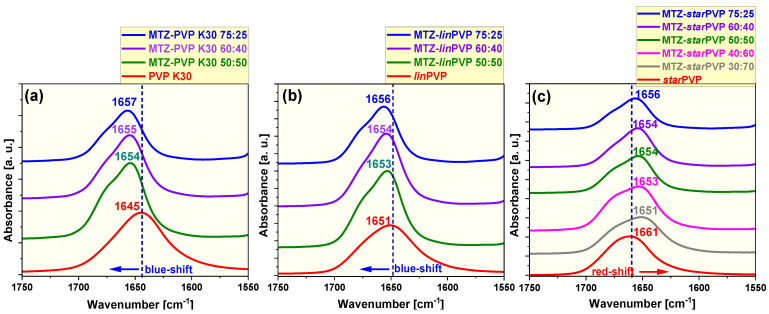
FTIR spectra of the carbonyl stretching region of (**a**) MTZ-PVP K30, (**b**) MTZ-*lin*PVP, and (**c**) MTZ-*star*PVP binary mixtures at different API–polymer ratios and that of the neat polymers, measured in the supercooled liquid/or glassy states.

We started the analysis of possible interactions between neat API and PVP samples from MTZ-PVP K30 BMs. As shown in Figure S8, MTZ in the solid dispersion shows a difference in spectral behavior compared to the molten state. It can be observed that the $\nu_{OH}$ band of MTZ in all binary systems (3700–3100 cm^−1^) is shifted to higher frequencies as well as considerably broadened and blurred relative to that in the liquid MTZ (3228 cm^−1^). The band originating from the C-H stretching vibrations of the H-C=C group located at 3103 cm^−1^ in the molten MTZ also shifts toward higher wavenumbers after mixing with PVP K30 (~3130 cm^−1^), which probably suggests the lack of C-H···O interactions involving a H-C=C moiety in the mixture. Moreover, other MTZ bands occurring at lower frequency ranges of BMs are also slightly shifted compared to those in neat API. All these facts confirm complete destruction of the crystal structure of the API in these mixtures. However, the most interesting feature of the studied MTZ-PVP K30 spectra is the behavior of the $\nu_{C=O}$ band of the polymer when mixed with API. As shown in Figure 3a, the $\nu_{C=O}$ peak of neat PVP-K30 located at 1645 cm^−1^ exhibits a blue shift (shift to higher wavenumbers) with the increasing content of MTZ in BMs (50:50 *w*/*w* 1654 cm^−1^; 60:40 *w*/*w* 1655 cm^−1^, 75:25 *w*/*w* 1657 cm^−1^). This fact suggests that the interaction between MTZ and PVP is a dipole–dipole intermolecular interaction, which intensifies with a higher amount of API. A similar interaction mechanism was observed for the ketoconazole-PVP and MK-0591/PVP systems [62,65,66]. In the case of the ketoconazole–PVP mixture, molecular calculations confirmed that ketoconazole has a dipole moment that is quite large in magnitude and may, therefore, interact strongly with other polar molecules such as PVP [62,65]. Since MTZ is a polar substance, one can expect a similar pattern of behavior.

Analyzing the spectra measured for MTZ-PVP K30 mixtures after the recrystallization process, it can be seen that the peaks related to the MTZ molecule show very similar spectral parameters (position, width, and intensity) in all studied BMs compared to these for neat crystalline APIs (Figure S9a). It indicates that MTZ occurs in the same crystalline form in the commercial sample and when mixed with PVPs. It should also be mentioned that the spectra of MTZ-*lin*PVP systems are very similar to those of MTZ-PVP K30 and reveal the same spectral effect for disordered and crystallized systems (Figures S8b and S9b). The $\nu_{C=O}$ band of *lin*PVP also shifts toward higher wavenumbers (blue shift) after mixing with polymers (50:50 *w*/*w* 1653 cm^−1^, 60:40 *w*/*w* 1654 cm^−1^, 75:25 *w*/*w* 1656 cm^−1^), indicating the dipole–dipole nature of intermolecular interactions between API and *lin*PVP (Figure 3b).

During further analysis, we compared the IR spectra of MTZ-*star*PVP (75:25, 60:40, 50:50, 40:60, 30:70 *w*/*w*) systems in the supercooled liquid/glassy states with those of neat components. As shown in Figure S8c, the $\nu_{OH}$ band of MTZ in solid dispersions is blue-shifted (3700–3100 cm^−1^) relative to that in the molten MTZ (3228 cm^−1^). Moreover, with the increase in API content in these systems, the $\nu_{OH}$ band is located close to that of neat MTZ. Similar to linear PVP samples, the peak associated with the C-H stretching vibrations of the H-C=C group occurs at a higher frequency value than that of neat API. Surprisingly, the introduction of MTZ in the *star*PVP matrix causes a considerable red shift of the $\nu_{C=O}$ band (shift to lower wavenumbers from 1661 cm^−1^ for neat *star*PVP to 1651 cm^−1^ for 30:70 *w*/*w* mixtures) due to the formation of hydrogen bonding (HB) between the hydroxyl group of MTZ and the carbonyl group of PVP (Figure 3c). It is worth noting that HB interactions are typically detected between APIs and polymers in BMs, as reported extensively, for example, between ibuprofen and PVP [67,68], indomethacin and PVP [69], esomeprazole and hydroxypropyl methylcellulose (HPMC) [70], flurbiprofen and poly(ethylene oxide) [71], and nifedipine and Eudragit^®^ [72], indicating that this is a key mechanism in the successful formation of amorphous or semi-crystalline solid dispersions [73]. It can also be mentioned that similar spectral effects related to the influence of PVP on API behavior were detected for MTZ-*star*PVP BMs in the recrystallized samples (Figure S9c). Also, in these binary systems, MTZ occurred in the same crystalline form as the neat initial sample.

In the next step, the IR spectra measured for BMs of MTZ with different PVPs, K30, *lin*PVP, and *star*PVP (75:25, 60:40, and 50:50 *w*/*w*), in the supercooled liquid and crystalline states were compared (Figures S10 and S11). As can be seen, no differences were essentially observed for these spectra, i.e., the subtle variations in the $\nu_{OH}$ band region may result from various water contents (always present in PVP) in the analyzed samples. It suggests that despite the difference in API–polymer interaction mechanisms in the mixtures, i.e., dipole–dipole (linear PVP) vs. HB forces (*star*PVP), the average interaction strength between MTZ and PVP samples is nearly the same.

Overall, based on the above FTIR spectroscopy findings, it can be concluded that the topology of PVP, linear or star-shaped, can significantly impact the drug–polymer interaction mechanism and then the API-EXC miscibility. For linear PVP samples (K30, *lin*PVP), dipole–dipole interactions occur/prevail, as was suggested by the blue shift of the $\nu_{C=O}$ band for BMs. Therefore, there is a weaker miscibility of PVP K30 and *lin*PVP with MTZ. In the case of *star*PVP, hydrogen bonding is the prime mechanism for the interaction of API and a polymer. As a result, MTZ is miscible with *star*PVP in a wider range of concentration, which is demonstrated by the red shift of the $\nu_{C=O}$ band in the FTIR spectra of solid dispersions. It should be noted that due to the similar M_n_ (~40,000 g/mol) of the PVPs used, one can suppose that the observed differences in solubility/miscibility with MTZ are associated with the topology of macromolecules.

Noticing variations in the interactions between the API and the polymer matrix, we decided to conduct further investigations of binary systems. To study the influence of the given PVP on the thermal properties, phase or glass transitions, and the crystallization rate of MTZ, systematic DSC measurements were performed on the prepared binary formulations characterized by varying contents of API.

### 3.4. Differential Scanning Calorimetry (DSC) data

**Figure 4:**
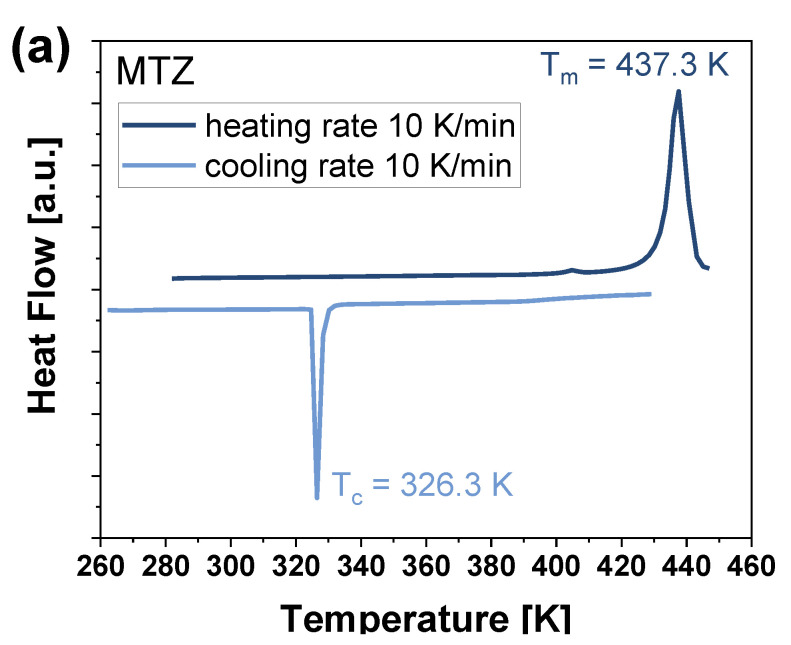

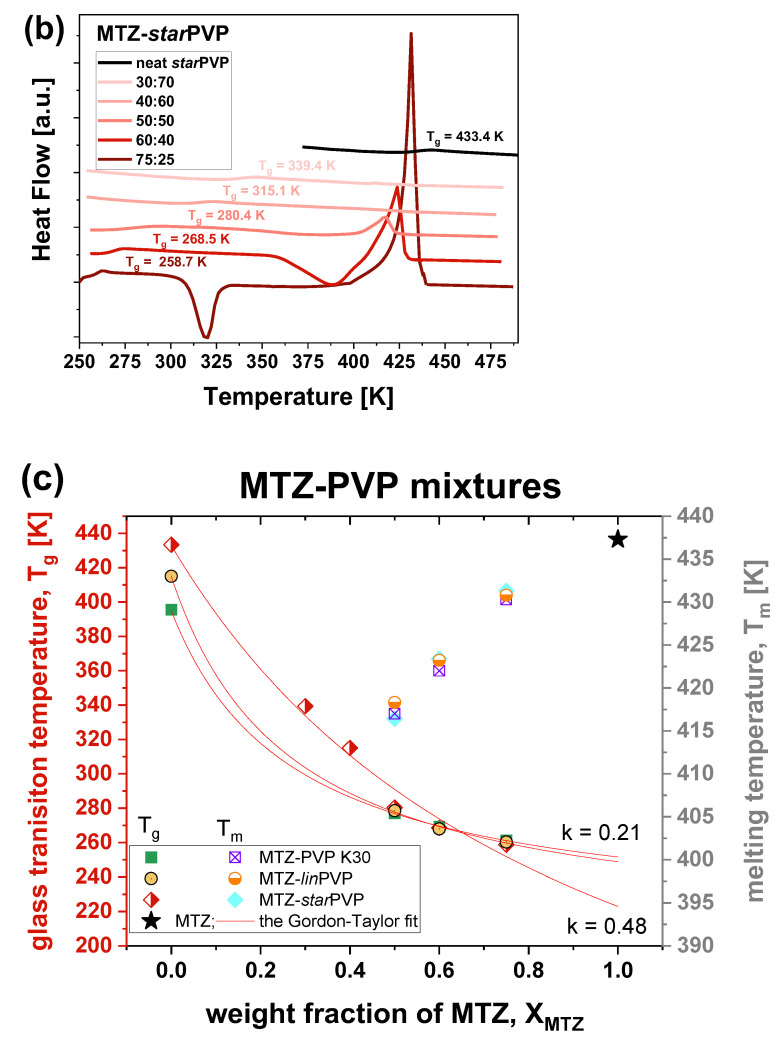
(**a**) DSC thermogram (ϕ = 10 K/min) of neat MTZ; (**b**) DSC thermograms (ϕ = 10 K/min) of neat *star*PVP and MTZ-*star*PVP mixtures with different weight ratios; (**c**) The dependency of calorimetric T_g_ and T_m_ versus weight fraction of MTZ for the examined binary systems. The solid red lines represent fits using the Gordon–Taylor equation (Equation (1)).

We started with DSC experiments of neat MTZ, and the obtained results are presented in Figure 4a. As can be seen, during the heating of crystalline API (at ϕ = 10 K/min), an endothermic peak at T = 437.3 K occurs in the thermogram (a dark blue line). Upon cooling the molten sample (with the same ϕ = 10 K/min), a single exothermic peak with a maximum at T = 326.3 K is observed (a light blue line). According to previous literature reports [44,74,75,76], the endothermic event at higher T corresponds to the melting of the API, while the exothermic one at lower T is attributed to the crystallization of MTZ. At this point, it is worth mentioning that inspired by studies demonstrating that the cooling rate of a sample significantly influences the phase transitions and makes it possible to transform the crystalline API into the amorphous form [77,78,79], we have made attempts to supercool/vitrify MTZ at various ϕ. Unfortunately, our efforts were unsuccessful. This implies that MTZ has an exceptionally low glass-forming ability, and it is not feasible to obtain it in the amorphous form using the standard vitrification method. However, in the system with two newly synthesized PVP polymers, linear and star-shaped, and a commercial sample PVP K30 (75:25, 60:40, 50:50 *w*/*w*), MTZ formed homogeneous amorphous mixtures. Therefore, we performed DSC measurements on these systems. Moreover, because *star*PVP has the ability to mix with the API in a higher range compared to PVPs with linear topology (PVP K30, *lin*PVP), which was mentioned in the previous section, we conducted DSC experiments on two additional MTZ-*star*PVP systems with the predominance of the polymer, i.e., 40:60 and 30:70 *w*/*w*. The main purpose of these investigations was to check how the addition of PVP with different topologies and weight percents will affect the glass-forming ability of MTZ, its physical stability, and the rate of recrystallization.

In Figure 4b, representative thermograms collected during the heating of amorphous API-*star*PVP ASDs with a rate of 10 K/min are presented. As can be seen, with increasing polymer content in solid dispersions, the T_g_ value increases. On the other hand, the peaks corresponding to the crystallization and melting processes gradually diminish/disappear. Interestingly, in the case of MTZ-*star*PVP in 40:60 and 30:70 *w*/*w* formulations, a complete suppression of the melting and crystallization processes is visible (only a thermal event connected to the glass transition can be detected). The values of T_g_ and T_m_ obtained for all examined systems (those determined for two other BMs, i.e., MTZ-PVP K30 and MTZ-*lin*PVP, have been given in Tables S1 and S2 in the SMs) are presented versus the weight fraction of MTZ (X_MTZ_) in Figure 4c. Analyzing the obtained data, one can conclude that depending on the content of PVP in ASDs, the T_m_s change significantly (up to 20 K). This may indicate substantial interactions between MTZ and the used polymeric matrices. Importantly, this conclusion agrees very well with the outcome of the FTIR analysis described above. Furthermore, other publications also suggest that the most likely explanation for the observed differences in T_m_ values of BMs appears to be the interactions occurring between the API and the polymer. For example, Kozyra et al. proposed such a scenario for BMs of itraconazole with three polymers, Eudragit L100, Carbopol 981, and hypromellose acetate succinate [80], while Chmiel et al. did so for nimesulide–Kollidon VA64 solid dispersions [81].

Furthermore, we can notice that the values of both T_g_ and T_m_ are independent of the type of PVP applied in a binary system. To characterize T_g_ vs. X_MTZ_ plots determined for each ASD and simultaneously estimate the T_g_ of MTZ, the Gordon–Taylor equation was applied [82]:

(1) $$T_{g}=\frac{X_{1}T_{g1}+kX_{2}T_{g2}}{X_{1}+{kX}_{2}}$$

where $T_{g1}$ and $T_{g2}$ are the glass transition temperatures (the subscript 1 refers to the component of the mixture with the lower $T_{g}$), $X_{1}$ is the weight fraction of component 1, while k is the constant, which represents a fitting parameter characterizing the curvature of the $T_{g}$ vs. $X_{1}$ evolution. In our studies, the data presented in Figure 4c ($T_{g}$ vs. X_MTZ_ plots) were analyzed using Equation (1) with the $T_{g}$ of API being a fitting parameter. As illustrated, the value of k determined for the MTZ-*star*PVP system (k = 0.48) is greater compared to the one obtained for MTZ-PVP K30 and MTZ-*lin*PVP ASDs (k = 0.21). There is also a slight difference (~5 K) in the $T_{g}$ of MTZ estimated from this analysis. The observed difference might be due to the fact that for the BM with the star-shaped polymer, we added two additional points at a higher polymer concentration. For other systems studied herein, it was not possible.

Noticing that MTZ-PVP 75:25, 60:40, and 50:50 *w*/*w* ASDs crystallize, we subsequently decided to perform non-isothermal DSC measurements to determine the activation energy (E_a_) for the crystallization of API in formulations with different PVPs.

**Figure 5:**
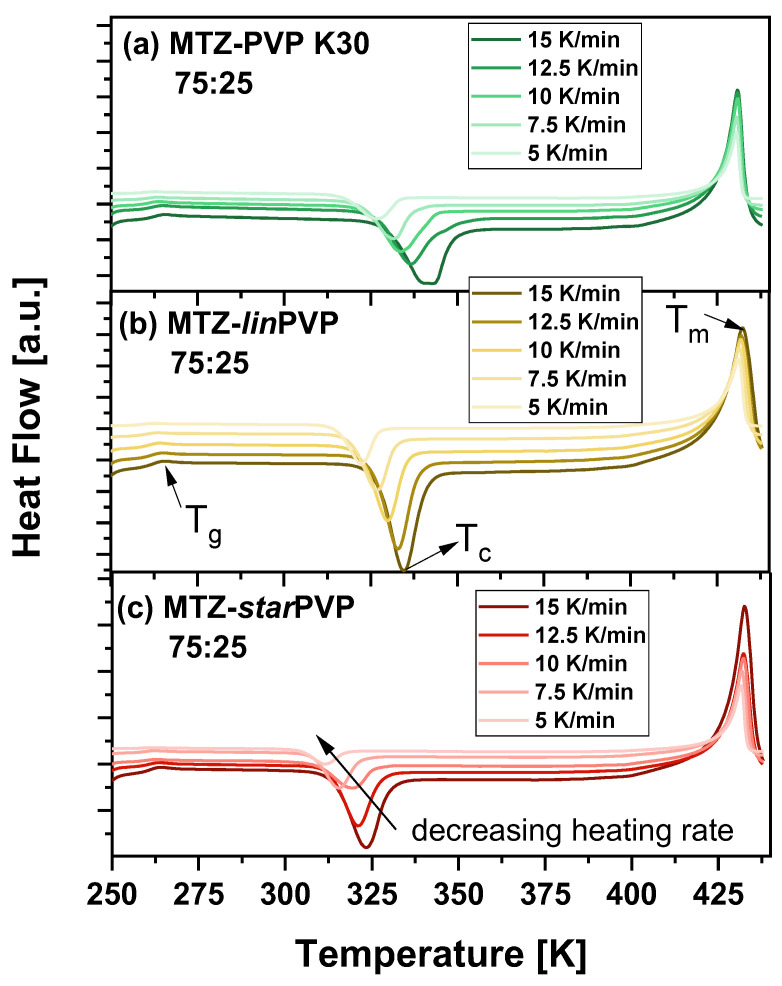
DSC thermograms for non-isothermal measurements of 75:25 *w*/*w* binary mixtures: (**a**) MTZ-PVP K30, (**b**) MTZ-*lin*PVP, (**c**) MTZ-*star*PVP.

**Figure 6:**
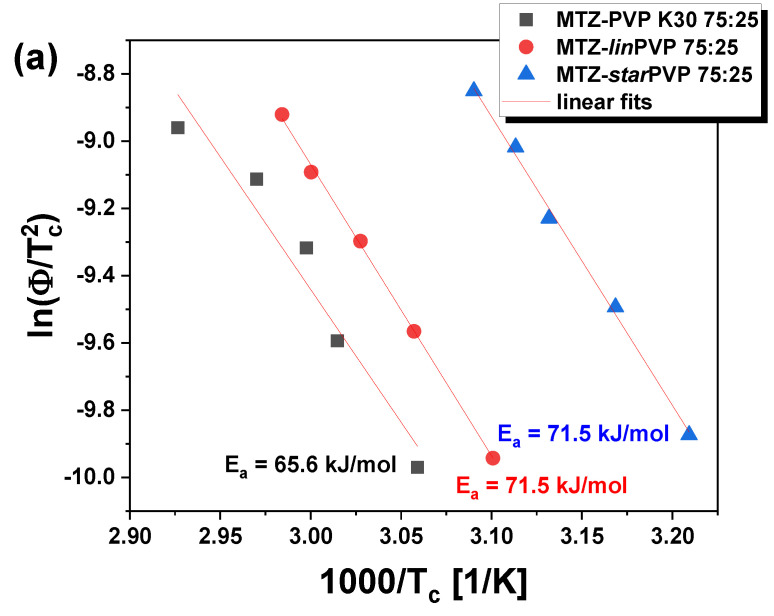

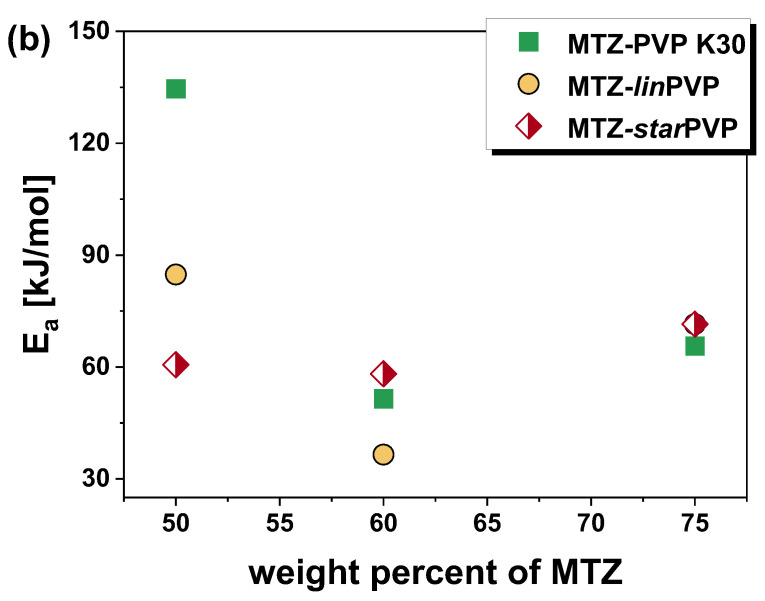
(**a**) Kissinger plots for the exothermic crystallization peaks in the representative MTZ-PVP 75:25 *w*/*w* ASDs. The solid red lines represent linear fits; (**b**) The dependency of E_a_ versus weight percent of MTZ in the examined binary systems.

Figure 5 shows representative thermograms for the freshly prepared mixtures that crystallize most rapidly, i.e., MTZ-PVP 75:25 *w*/*w*. Note that the same measurements (with various ϕ ranging from 2.5 to 15 K/min) were performed for other API-PVP systems, i.e., 60:40 and 50:50 *w*/*w*. The values of crystallization temperatures (T_c_) for all studied BMs, prepared at various weight ratios, are presented in tabular form in the SMs (see Tables S3–S5). As can be observed in Figure 5, three thermal events are visible during each scan. Except for two endothermic peaks occurring at the lowest and highest T (attributed to the glass transition and the melting, respectively), there is an exothermic peak corresponding to the crystallization of MTZ. It is visible that in the case of each MTZ-PVP system, the T_c_ values (the peak temperature of crystallization) increase with increasing ϕ. In turn, the T_g_s and T_m_s do not significantly vary depending on the heating rate. The analysis of non-isothermal DSC data obtained for all studied systems (Figure 6a) used the Kissinger method [83]:

(2) $$\ln\left(\frac{\phi }{T_{c}}\right)=C_{k}-\frac{E_{a}}{RT_{c}};$$

where $C_{k}$ is a fitting parameter and R is a gas constant, enabling us to calculate the activation energy (E_a_) of the crystallization of MTZ in various ASDs.

It is well observed that for the MTZ-PVP 75:25 *w*/*w* BMs, the values of the E_a_ for the API crystallization are very similar (65.6–71.5 kJ/mol). The lowest E_a_ (65.6 kJ/mol) was determined for the mixture with commercial PVP (PVP K30). However, as the polymer content increases in the system, the differences in crystallization energies become greater. Interestingly, in the case of MTZ-PVP 50:50 *w*/*w* BMs, E_a_ changes as follows: E_aMTZ-PVPK30_ > E_aMTZ-*lin*PVP_ > E_aMTZ-*star*PVP_.

In the last step of calorimetric studies, we conducted isothermal crystallization measurements at T = 295 K for MTZ-PVP 75:25 *w*/*w* binary mixtures. Herein, it should be mentioned that we selected only systems with such content of both components because they crystallized quickly at this temperature. In other BMs, the crystallization was much longer. Therefore, such studies were not performed.

**Figure 7:**
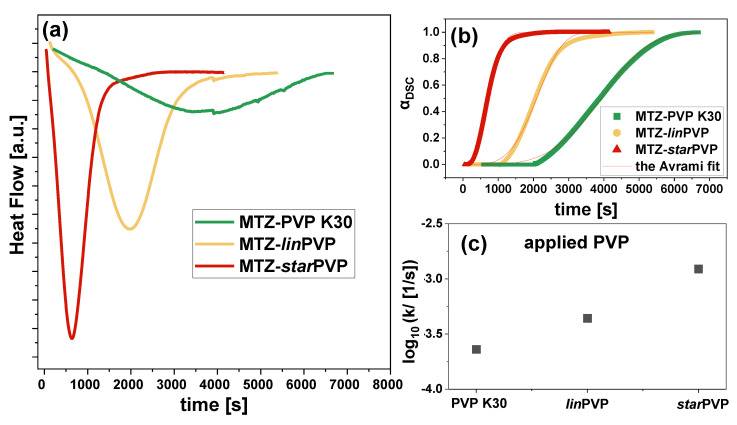
(**a**) DSC thermograms of isothermal measurements (T = 295 K) for MTZ-PVP 75:25 *w*/*w* mixtures; (**b**) The progress of the crystallization process carried out for the examined MTZ-PVP systems. Solid lines represent Avrami fits; (**c**) Crystallization rate constants (k) of MTZ depending on the applied PVP in BMs.

In Figure 7a, we plotted the heat released during the crystallization process versus time. At first sight, it can be seen that the time scale of crystallization of MTZ in various PVP matrices is different. To quantify this observation, the following formula was applied:

(3) $$\alpha_{DSC}=\frac{\Delta H(t)}{{\Delta H}_{total}}$$

where $\alpha_{DSC}$ is the progress of crystallization, while ΔH(t) and ${\Delta H}_{total}$ mean the enthalpy variation at different times and the total heat of the crystallization, respectively. With the crystallization kinetic curves constructed (see Figure 7b), we described them using the Avrami model:

(4) $$\alpha \left(t\right)=1-exp(-kt^{n})$$

where k is the rate constant of crystallization and n is the Avrami exponent, which is generally assigned to a dimension of growing crystals. As can be deduced from Figure 7c, there is a clear variation in the constant rate of MTZ crystallization in the studied systems. Importantly, a commercial PVP K30 turned out to be the most effective polymer in slowing down API crystallization. On the other hand, this process was the fastest in the binary system with *star*PVP. Herein, it is worth noting that although Figure 6b shows very similar (within the measuring error) values of the E_a_ for the MTZ crystallization in all 75:25 *w*/*w* BMs, isothermal DSC measurements (T = 295 K) for the same binary systems revealed that the crystallization rate constants (k) of API vary significantly depending on the polymer used. According to literature reports, the crystallization rate has a clear correlation with the activation energy of this process. Generally, k increases with a decrease in E_a_ [84,85]. However, it should be emphasized that the crystallization rate is also significantly influenced by the viscosity of the mixtures, molecular interactions, and the degree of cooling. This may be the reason for the lack of correlation between both parameters in the case of examined BMs.

To explain the data presented and discussed above, one can suppose that the substantial difference in the activation barrier and the constant rate of crystallization of MTZ from BMs with various PVPs might be due to different intermolecular interactions occurring between both components in solid dispersions, as concluded from FTIR measurements. However, there is also another possibility that the API in binary systems crystallizes into a different polymorphic form. Although the second scenario is less probable since FTIR investigations indicated that, irrespective of the macromolecule, MTZ forms the same crystals, we decided to verify this hypothesis as well. For that purpose, XRD studies on BMs composed of API and three PVP polymers mixed in different weight ratios were performed.

### 3.5. X-ray Diffraction (XRD) Data

**Figure 8:**
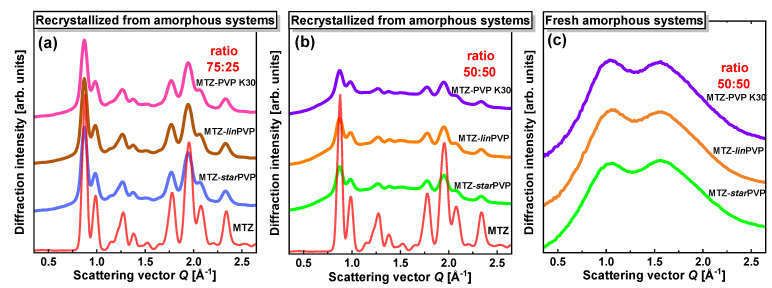
XRD patterns of neat MTZ and its solid dispersions with different PVP polymers: (**a**) in 75:25 (*w*/*w*) ratio after recrystallization, (**b**) in 50:50 (*w*/*w*) ratio after recrystallization, and (**c**) in 50:50 (*w*/*w*) ratio for fresh amorphous systems.

**Figure 9:**
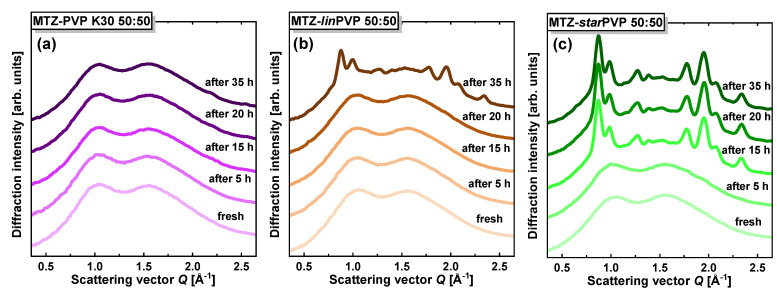
The temporal evolution of the XRD patterns for freshly prepared MTZ-PVP 50:50 *w*/*w* binary systems: (**a**) MTZ-PVP K30, (**b**) MTZ-*lin*PVP, and (**c**) MTZ-*star*PVP.

XRD patterns of MTZ-PVP BMs prepared in two weight ratios, 75:25 and 50:50, are illustrated in Figure 8a and Figure 8b, respectively. They were collected for systems in which MTZ recrystallized (after around one day after preparation). For all cases, despite the different PVP topologies, it can be seen that MTZ recrystallized to the same polymorphic form as crystalline MTZ (the diffractogram of neat crystalline API was set together for comparison). The crystal structure of this MTZ form belongs to the monoclinic $P2_{1}/c$ space group with lattice parameters a ≈ 7.03 Å, b ≈ 8.73 Å, c ≈ 12.82 Å, and cell angles α ≈ 90°, β ≈ 94.5°, γ ≈ 90° (CCDC number 1212679 in Cambridge Crystallographic Data Centre). Moreover, Figure 8c demonstrates the XRD data collected for freshly prepared API-PVP 50:50 *w*/*w* binary systems. They are very similar to each other, exhibiting two broad diffraction maxima at nearly the same positions. The recording of amorphous diffractograms for 75:25 *w*/*w* BMs was impossible due to the fast recrystallization of MTZ. Further, temporal XRD studies of the MTZ-PVP 50:50 *w*/*w* solid dispersions were performed, starting from the freshly prepared amorphous systems. The results presented in Figure 9 indicate that the physical stability of MTZ in these mixtures is affected by the topology of PVP. MTZ in the BM with *star*PVP recrystallized fully after 15 h; in the BM with *lin*PVP, MTZ started to recrystallize later—after 35 h; while in the system with PVP K30, it was still stable in the amorphous form after 55 h.

**Figure 10:**
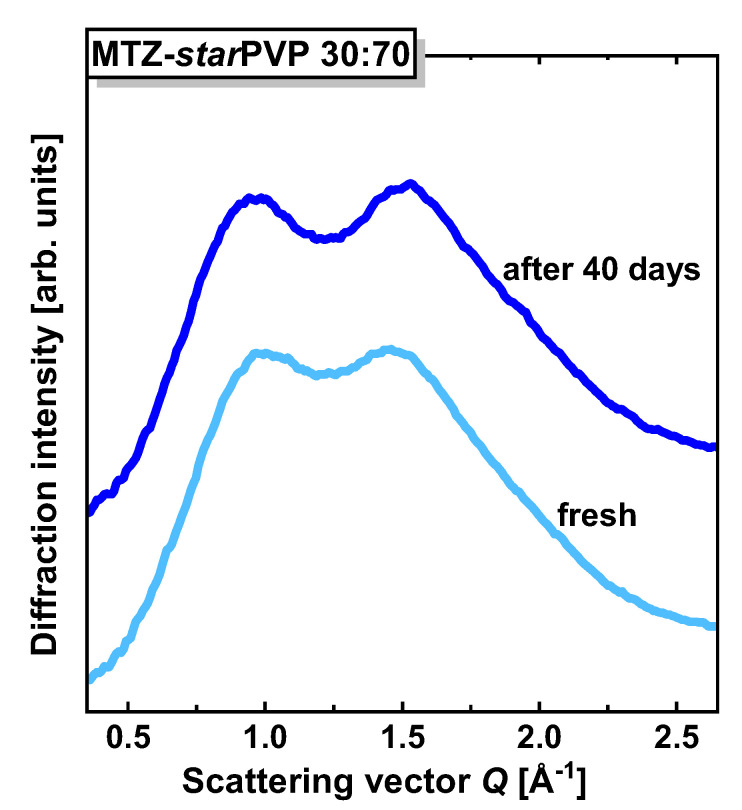
XRD patterns of the MTZ-*star*PVP 30:70 *w*/*w* binary mixture for a fresh sample (light blue line) and after 40 days of storage (dark blue line).

Herein, it is worth emphasizing that we also conducted preliminary long-term XRD studies for the MTZ-*star*PVP system containing the highest possible amount of the matrix (i.e., 70 wt%). This research showed that the recrystallization of API was strongly inhibited, and the amorphous form of MTZ was stable for over one month (see Figure 10). This is evidenced by exactly the same pattern of diffractograms for both samples, i.e., the fresh BM measured immediately after preparation and the formulation stored for over a month at room temperature.

Thus, the obtained data indicated that with respect to the linear PVPs of similar M_n_, the star-shaped macromolecule is the least effective in stabilizing amorphous APIs at a similar range of concentrations (50:50, 75:25 *w*/*w*). However, at a higher star-shaped polymer content in BMs, which is not attainable for the linear PVP due to weaker miscibility, the crystallization of MTZ is strongly suppressed and not detectable for more than one month.

## 4. Conclusions

To sum up, we successfully obtained novel PVP homopolymers with different architectures (linear and three-arm star) using thermally initiated RAFT processes. Hence, three polymers, (i) PVP K30 (M_n_ = 40,500 g/mol, Ð = 2.05), (ii) *lin*PVP (M_n_ = 37,600 g/mol, Ð = 1.22), and (iii) *star*PVP (M_n_ = 38,700 g/mol, Ð = 1.48) with similar molecular weights but varying dispersities and topologies were employed to create BMs with a fast-crystallizing drug—MTZ. Our main goal was to investigate whether the polymer architecture and/or macrostructural parameters (Ð), as well as the PVP content in the mixtures, influence the intermolecular interactions, physical stability, and the rate of MTZ recrystallization. The first remarkable effect observed during the samples’ preparation was the enhanced miscibility of the API with star-shaped polymers compared to the linear-synthesized and commercial PVP. Interestingly, FTIR studies indicated that this may be related to a different interaction mechanism between various PVPs and examined pharmaceutical. As demonstrated, dipole–dipole interactions dominate between linear polymers (PVP K30, *lin*PVP) and MTZ, while interactions between the star-shaped macromolecule (*star*PVP) and the API primarily occur through hydrogen bonds. In turn, detailed isothermal DSC studies on 75:25 *w*/*w* systems allowed us to conclude that among the tested polymer matrices, PVP K30 most effectively retards the crystallization of MTZ, while the *star*PVP exhibits the weakest ability to inhibit this process effectively. These results were also confirmed by XRD studies performed on MTZ-PVP BMs with 50 wt% of API. Moreover, non-isothermal DSC measurements revealed differences in the activation barrier for API crystallization depending on the topology of the polymer and its content in solid dispersions. Additionally, FTIR and XRD investigations indicated that in BMs with all examined PVPs, MTZ recrystallizes into the same polymorphic form. Surprisingly, the new polymer material, *star*PVP, turned out to have the ability to strongly inhibit the recrystallization of MTZ in a mixture containing a high percentage of the polymer (i.e., 70 wt%, which is impossible to achieve for linear PVPs). For this binary system (MTZ-*star*PVP 30:70 *w*/*w*), we observed the stability of amorphous MTZ for over one month.

## Data Availability

Data are contained within the article and Supplementary Materials.

## Supplementary Materials

The following are available online at https://www.mdpi.com/article/10.3390/pharmaceutics16010136/s1, Figure S1: ^1^H NMR spectrum of trifunctional CTA2 in CDCl_3_ (500 MHz); Figure S2: ^1^H NMR spectrum of *lin*PVP in CDCl_3_ (600 MHz); Figure S3: ^13^C NMR spectrum of *lin*PVP in CDCl_3_ (600 MHz); Figure S4: SEC traces of PVP samples: commercial (PVP K30 - grey line) and self-synthesized (*lin*PVP-red line, *star*PVP-blue line); Figure S5: ^1^H NMR spectrum of *star*PVP in CDCl_3_ (600 MHz); Figure S6: ^13^C NMR spectrum of *star*PVP in CDCl_3_ (600 MHz); Figure S7: Infrared spectra of crystalline and molten MTZ as well as PVP samples. Data were presented in two spectral regions: (a) 3800–2400 cm^−1^ and (b)1800–400 cm^−1^; Figure S8: Infrared spectra of (a) MTZ-PVP K30, (b) MTZ-*lin*PVP, and (c) MTZ-*star*PVP binary mixtures prepared in different weight ratios, measured at T=295 K (in the supercooled liquid/or glassy states) together with the spectra registered for the molten MTZ and neat PVP polymers, presented in the ranges of 3800–2400 cm^−1^ (left) and 1800–400 cm^−1^ (right); Figure S9: Infrared spectra of (a) MTZ-PVP K30, (b) MTZ-*lin*PVP, and (c) MTZ-*star*PVP binary mixtures (samples after recrystallization) prepared in different molar ratios together with the spectra registered for the crystalline MTZ and neat PVP polymers, presented in the ranges of 3800–2400 cm^−1^ (left) and 1800–400 cm^−1^ (right); Figure S10: Infrared spectra of binary mixtures of MTZ with all PVP samples (K30, *lin*PVP and *star*PVP) ((a) 75:25, (b) 60:40, (c) 50:50 *w*/*w*) measured in the supercooled liquid state, presented in the ranges of 3800–2400 cm^−1^ (left) and 1800–400 cm^−1^ (right); Figure S11: Infrared spectra of binary mixtures of MTZ with all PVP samples (K30, *lin*PVP and *star*PVP) ((a) 75:25, (b) 60:40, (c) 50:50 *w*/*w*) measured in the crystalline state, presented in the ranges of 3800–2400 cm^−1^ (left) and 1800–400 cm^−1^ (right); Table S1: The calorimetric glass transition temperature values (heating rate, ϕ = 10 K/min) for neat PVPs and examined MTZ-PVP binary mixtures in various weight ratios; Table S2: The calorimetric melting temperature values (ϕ = 10 K/min) for neat MTZ and the examined MTZ-PVP binary mixtures in various weight ratios; Table S3: The crystallization temperature values for MTZ-PVP K30 mixtures depending on the heating rate (ϕ); Table S4: The crystallization temperature values for MTZ-*lin*PVP mixtures depending on the heating rate (ϕ); Table S5: The crystallization temperature values for MTZ-*star*PVP mixtures depending on the heating rate (ϕ). Details concerning the synthesis of CTA2, *lin*PVP, and *star*PVP (including ^1^H NMR and ^13^C NMR spectra and SEC traces of PVP samples).
