# Supplementary material for: The Impact of Various Poly(vinylpyrrolidone) Polymers on the Crystallization Process of Metronidazole
Source: Pharmaceutics. 2024 Jan 19;16(1):136. doi: 10.3390/pharmaceutics16010136 (PMC10820696; doi:10.3390/pharmaceutics16010136)
Supplement: Supplementary file 1 [file pharmaceutics-16-00136-s001.zip › pharmaceutics-2826484-supplementary.pdf]

Supplementary Materials

The Impact of Various Poly(vinylpyrrolidone) Polymers on the Crystallization Process of Metronidazole

**Luiza Orszulak [1,*], Taoufik Lamrani [2], Magdalena Tarnacka [2], Barbara Hachuła [1], Karolina Jurkiewicz [2],
Patryk Zioła [2], Anna Mrozek-Wilczkiewicz [2,3], Ewa Kamińska [4] and Kamil Kamiński [2]**

[1] Institute of Chemistry, Faculty of Science and Technology, University of Silesia in Katowice, Szkolna 9, 40-007 Katowice, Poland; barbara.hachula@us.edu.pl
[2] Institute of Physics, Faculty of Science and Technology, University of Silesia in Katowice, 75 Pułku Piechoty 1A, 41-500 Chorzow, Poland; taoufik.lamrani@us.edu.pl (T.L.); magdalena.tarnacka@us.edu.pl (M.T.); karolina.jurkiewicz@us.edu.pl (K.J.); patryk.ziola@us.edu.pl (P.Z.); anna.mrozek-wilczkiewicz@us.edu.pl (A.M.-W.); kamil.kaminski@us.edu.pl (K.K.)
[3] Biotechnology Centre, Silesian University of Technology, Boleslawa Krzywoustego 8, 44-100 Gliwice, Poland
[4] Department of Pharmacognosy and Phytochemistry, Faculty of Pharmaceutical Sciences in Sosnowiec, Medical University of Silesia in Katowice, Jagiellonska 4, 41-200 Sosnowiec, Poland; ekaminska@sum.edu.pl
* Correspondence: luiza.orszulak@us.edu.pl

1. Experimental

1.1. Synthesis of trifunctional chain transfer agent (CTA2)

Trifunctional chain transfer agent (1,3,5-benzyl tri(diethyldithiocarbamate), CTA2) was prepared in a single-step route by the reaction of 1,3,5-tribromomethyl benzene with sodium diethyldithiocarbamate according to the following procedure. In a 100 mL two-neck round-bottom flask equipped with a magnetic stirrer, inlet-outlet nitrogen, and dropping funnel, sodium diethyldithiocarbamate trihydrate (2 g, 8.88 mmol) was dissolved in methanol (40 mL) under a nitrogen atmosphere and cooled to 273 K. A solution of 1,3,5-tribromomethyl benzene (1 g, 2.80 mmol) in methanol (10 mL) was added dropwise over a 30 min period. The reaction was gradually warmed to room temperature and remained under magnetic stirring for a further 72 h. Then, the yellowish precipitate was filtered, washed with cold methanol, and left to dry to constant mass. The structure of the obtained 1,3,5-benzyl tri(diethyldithiocarbamate) was confirmed by ^1H NMR spectrum (see **Figure S1**).

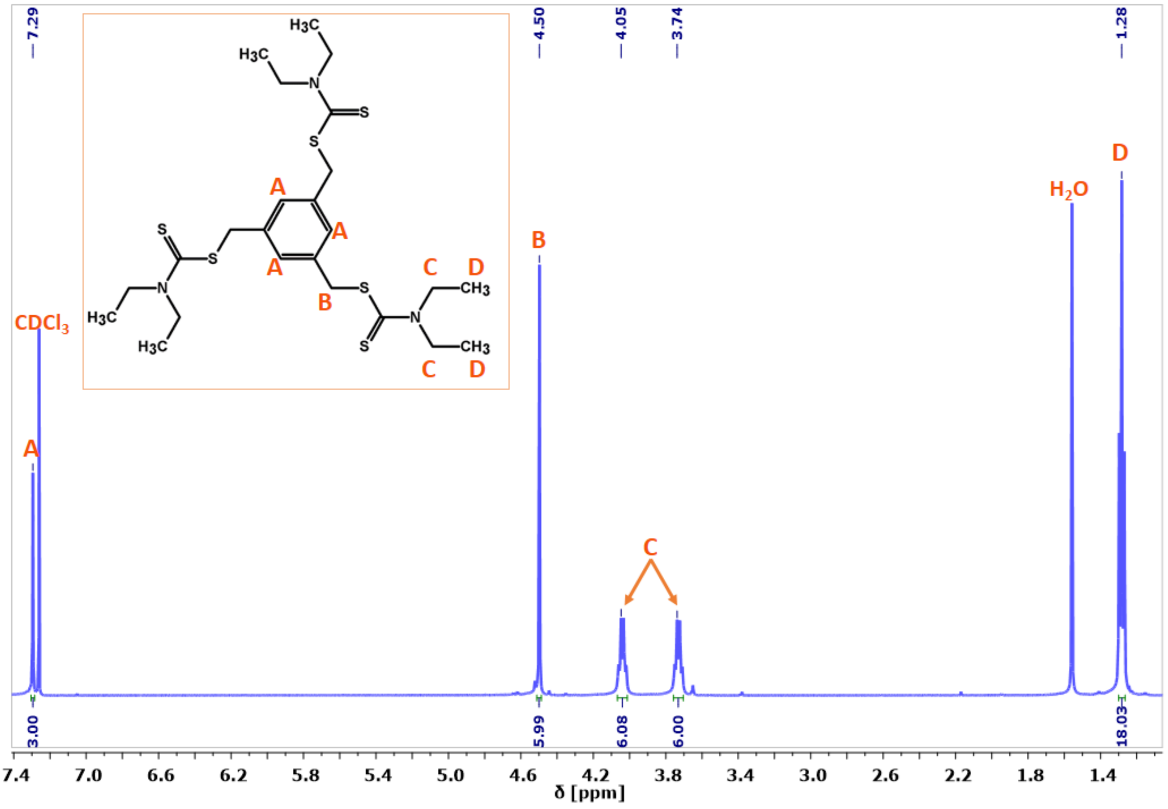

Figure S1. ^1H NMR spectrum of trifunctional CTA2 in $CDCl_3$ (500 MHz).

1.2. Synthesis of PVP with linear topology (linPVP)

Thermally-initiated Reversible Addition Fragmentation Chain Transfer (RAFT) polymerization of N-vinylpyrrolidone (VP) using CTA1 as a chain transfer agent and 2,2′-azobis(2-methylpropionitrile) (AIBN) as an initiator with molar ratios $[VP]_0/[CTA1]_0/[AIBN]_0$ = 500/1/0.25) was carried out as follows. Prior to polymerization, VP was passed through an alumina column to remove the inhibitor. CTA1 (33.5 mg, 0.15 mmol) and VP (8 mL, 74.86 mmol) were placed in a Schlenk flask with a magnetic stirring bar. The solution was purged under nitrogen and purified by three freeze-pump-thaw cycles. Then, the solution of AIBN (187 μL, 0.037 mmol) was added to the reaction mixture and the flask was immersed in an oil bath thermostated at 60 °C to start the reaction. The polymerization was quenched after a predetermined time (t = 3.5 h) by cooling and exposing the reaction mixture to air. The product was precipitated with cold diethyl ether and re-dissolved in chloroform and this cycle was repeated twice. The polymer was isolated, filtered, and then dried under vacuum to a constant mass. The structure of the obtained linear PVP was confirmed by ^1H and ^{13}C NMR spectra (see **Figures S2 and S3**). The molecular weight and dispersity of the produced *lin*PVP were determined by size exclusion chromatography (SEC) (see **Figure S4**).

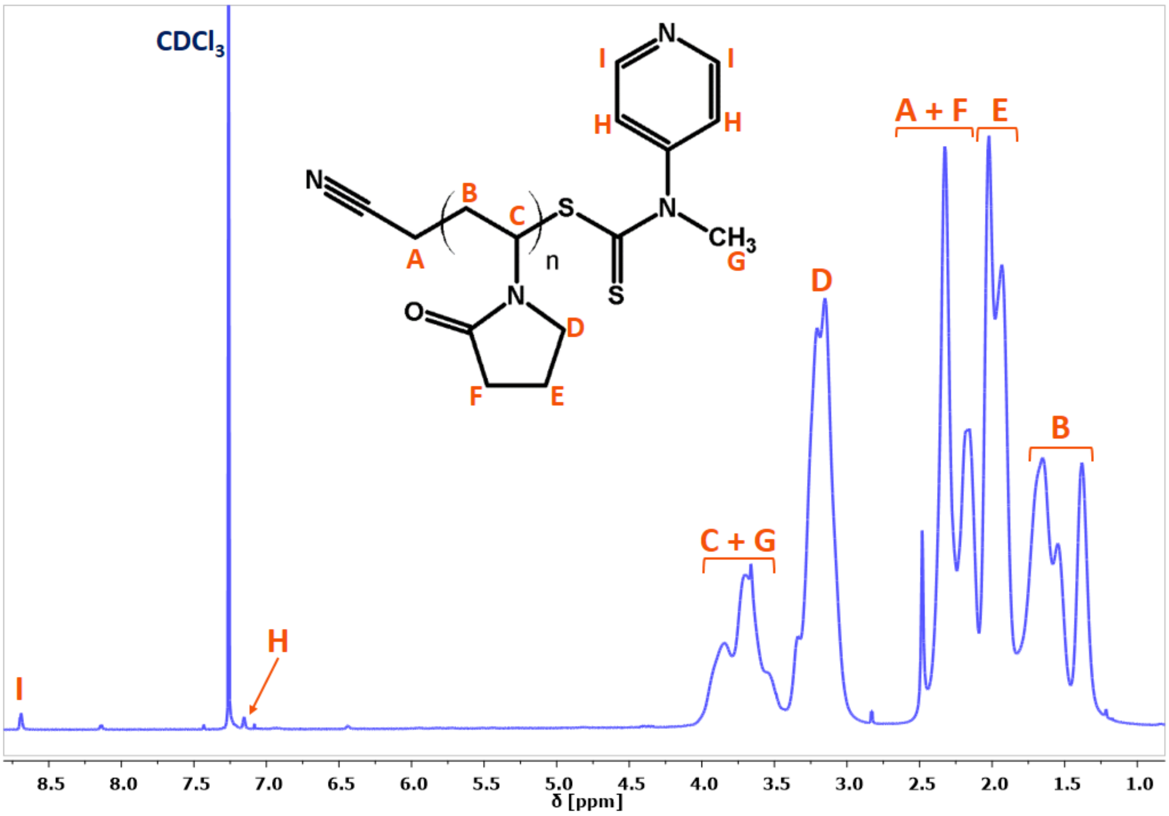

Figure S2. ^{1}H NMR spectrum of *lin*PVP in $CDCl_3$ (600 MHz).

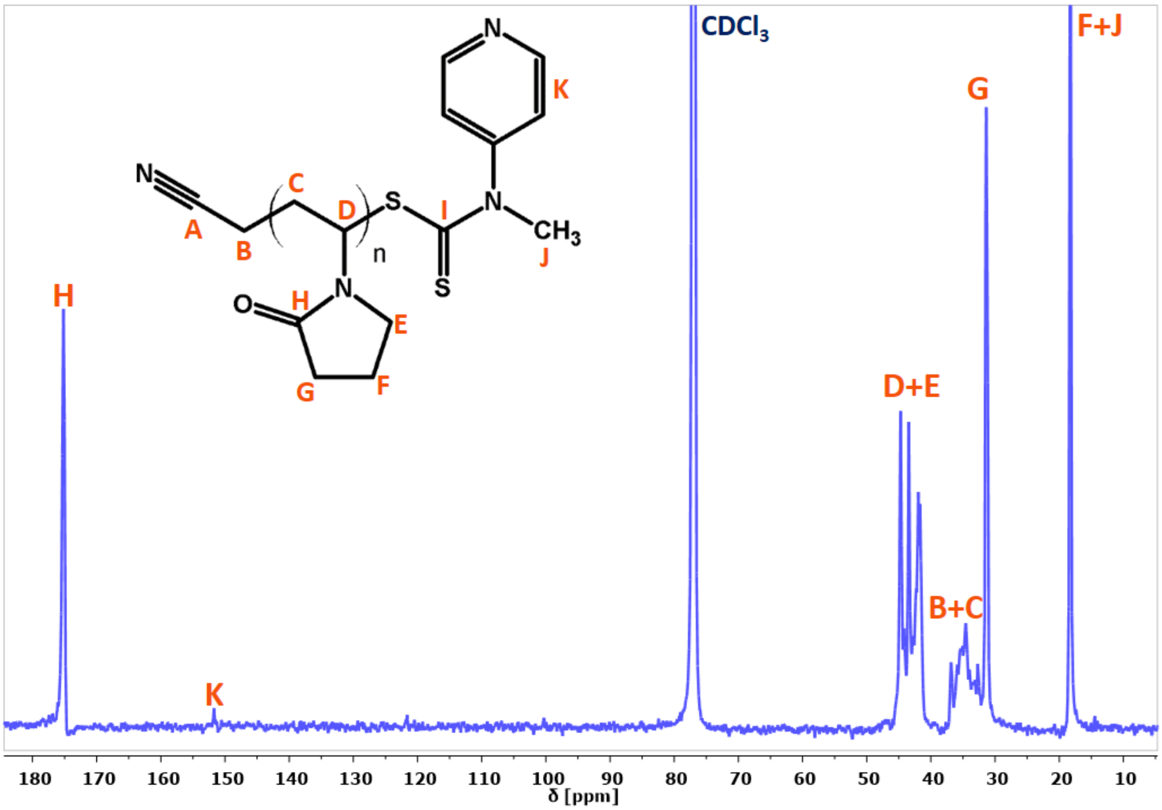

Figure S3. ^{13}C NMR spectrum of *lin*PVP in $CDCl_3$ (600 MHz).

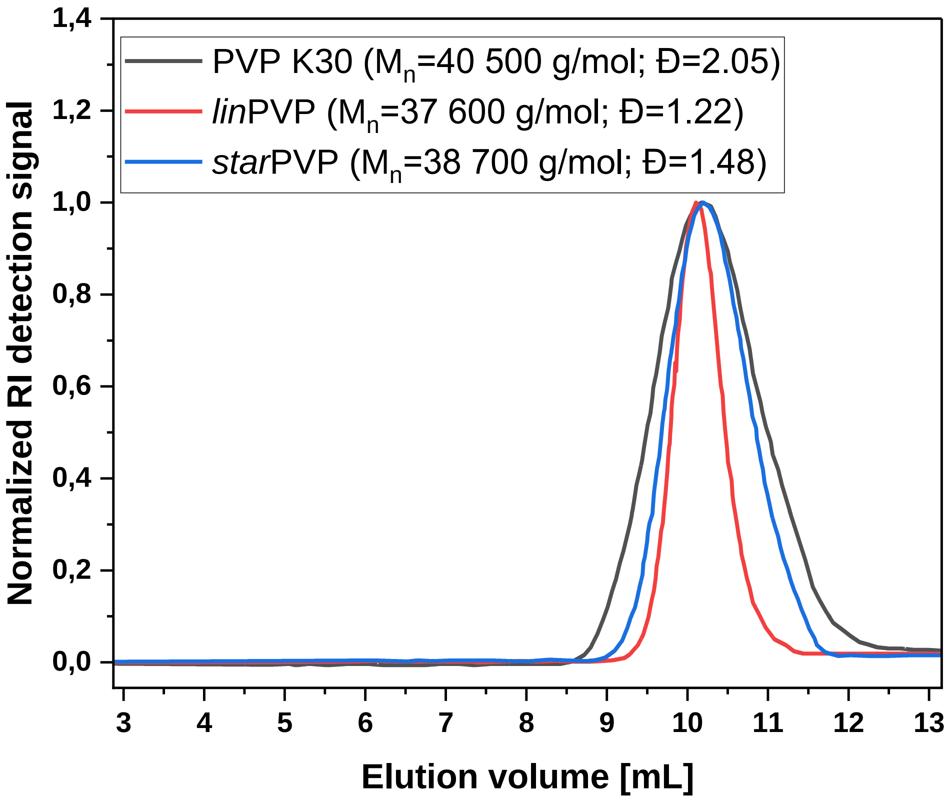

Figure S4. SEC traces of PVP samples: commercial (PVP K30 - grey line) and self-synthesized (*lin*PVP - red line, *star*PVP - blue line).

1.3. Synthesis of PVP with star topology (starPVP)

Thermally-initiated RAFT polymerization of VP using previously synthesized CTA2 as a chain transfer agent and AIBN as an initiator with molar ratios $[VP]_0/[CTA2]_0/[AIBN]_0$ = 400/5/1) was carried out as follows. Before polymerization, VP was passed through an alumina column to remove the inhibitor. CTA2 (0.13148 g, 0.23 mmol), VP (2 mL, 18.72 mmol) and DCM (4 mL, 200% v/v in relation to the monomer) were placed in a Schlenk flask with a magnetic stirring bar. The solution was purged under nitrogen and purified by three freeze-pump-thaw cycles. Then, the solution of AIBN (234 µL, 0.047 mmol) was added to the reaction mixture and the flask was immersed in an oil bath thermostated at 60 °C to start the reaction. The polymerization was quenched after a predetermined time (t = 8 h) by cooling and

exposing the reaction mixture to air. The product was precipitated with cold diethyl ether and re-dissolved in chloroform, and this cycle was repeated twice. The polymer was isolated, filtered, and then dried under vacuum to a constant mass. The structure of the obtained three-arm star-shaped PVP was confirmed by ^1H and ^{13}C NMR spectra (see **Figures S5 and S6**). The molecular weight and dispersity of the produced *star*PVP were determined by SEC (see **Figure S4**).

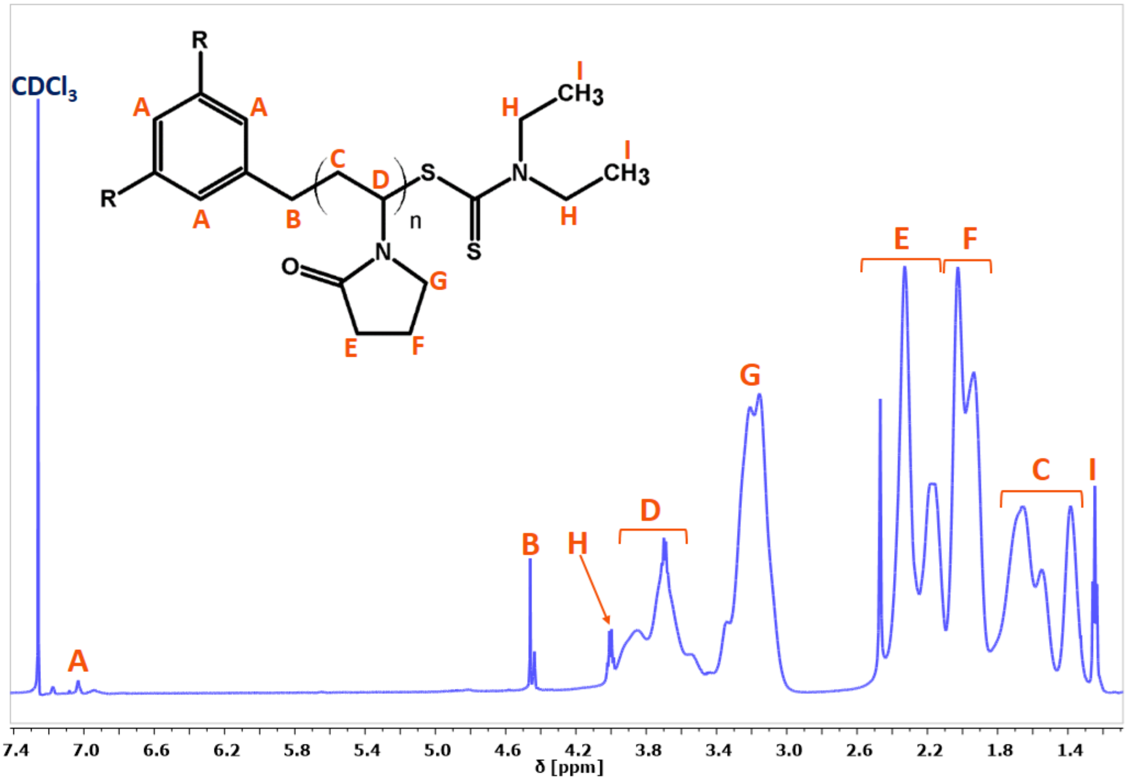

Figure S5. ^1H NMR spectrum of *star*PVP in $CDCl_3$ (600 MHz).

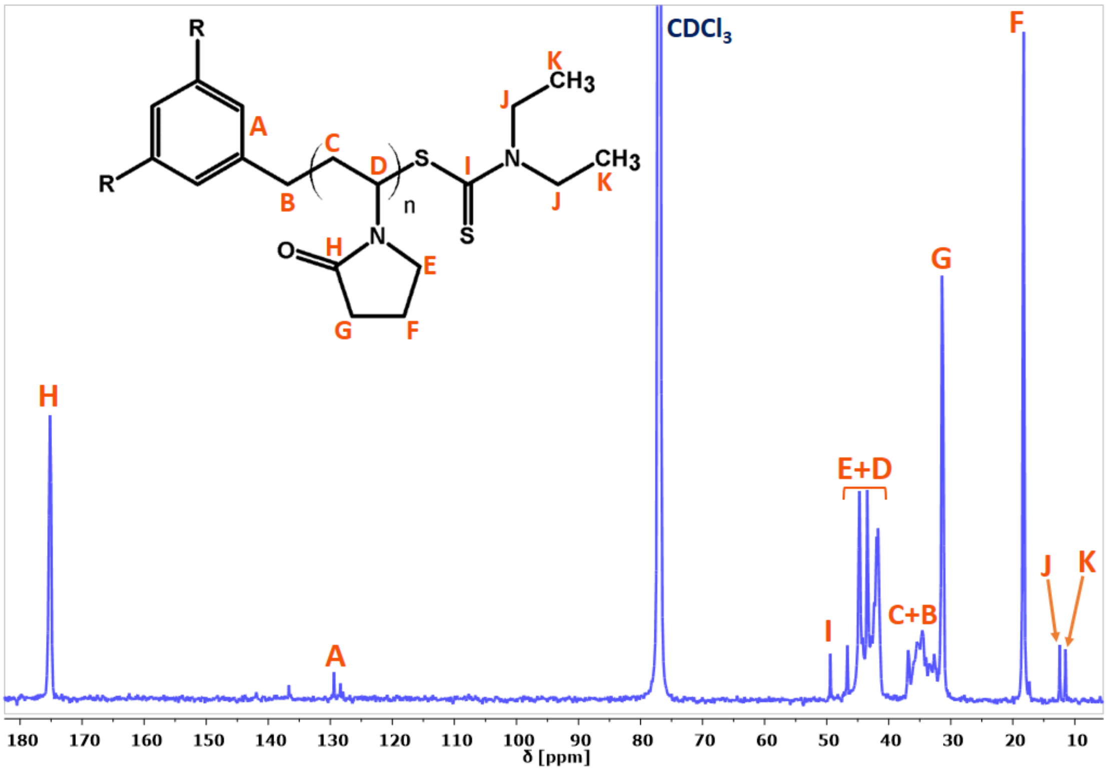

Figure S6. ^{13}C NMR spectrum of *star*PVP in $CDCl_3$ (600 MHz).

2. FTIR data

In the IR spectrum of crystalline MTZ, shown in **Figure S7**, one can see a sharp peak at 3099 cm^{-1} assigned to the CH stretching vibrations of the H-C=C group. The bands located from 3000 to 2800 cm^{-1} correspond to the aliphatic CH stretching vibrations of methyl and methylene groups. The absorption signals occurring at a lower frequency region at 1534 and 1367 cm^{-1} are related to the asymmetric and symmetric stretching vibrations of the NO_2 group, respectively. The peaks detected at 1472 and 1427 cm^{-1} originate from the mixed C=C and C=N stretching vibrations as well as the CH bending of methyl and methylene groups. At 1264 and 1185 cm^{-1}, there are the bands associated with in-plane bending of the CH group (H-C=C) and the C-N stretching (tertiary amine group) [1–3]. The peak due to the in-plane OH deformation and CH_2 twisting out-of-plane vibration occurs at 1073 cm^{-1}. The scissoring in-plane bending of NO_2 moiety gives rise to a band at 825 cm^{-1}. A peak at 743 cm^{-1} corresponds to the NO_2 wagging vibration.

IR spectra of PVP samples in the higher wavenumber range are characterized by the bands originating from the stretching vibrations of OH groups, because PVP is hygroscopic, (3700-3100 cm^{-1}) and CH groups (3100-2800 cm^{-1}). In more detail, the peak at 2952 cm^{-1} corresponds to the asymmetric stretching vibrations of the CH_2 groups of the pyrrole ring, whereas the signal at 2925 cm^{-1} is related to the symmetric stretching vibrations of the CH_2 groups of a chain [4]. The band located at about 1650 cm^{-1} (1645 cm^{-1} for PVP K30, 1651 cm^{-1} for *lin*PVP, 1661 cm^{-1} for *star*PVP) is associated with the stretching vibrations of C=O moiety (**Figure 2** in the main manuscript). The signals assigned to the bending vibrations of CH groups occur at 1461 and 1374 cm^{-1} [5]. The peak observed at 1287 cm^{-1} originates from the wagging vibrations of CH_2 moieties and the stretching vibrations of the C-N group [4]. At 1019 cm^{-1}, the band associated with the CH_2 rocking vibrations and the stretching vibrations of C-C moieties is observed. The peak at 934 cm^{-1} corresponds to the stretching vibrations of C-C groups, while that at 845 cm^{-1} is related to the bending vibrations of CH_2 moieties. The signal at 572 cm^{-1} is assigned to the bending vibrations of the N-C=O group.

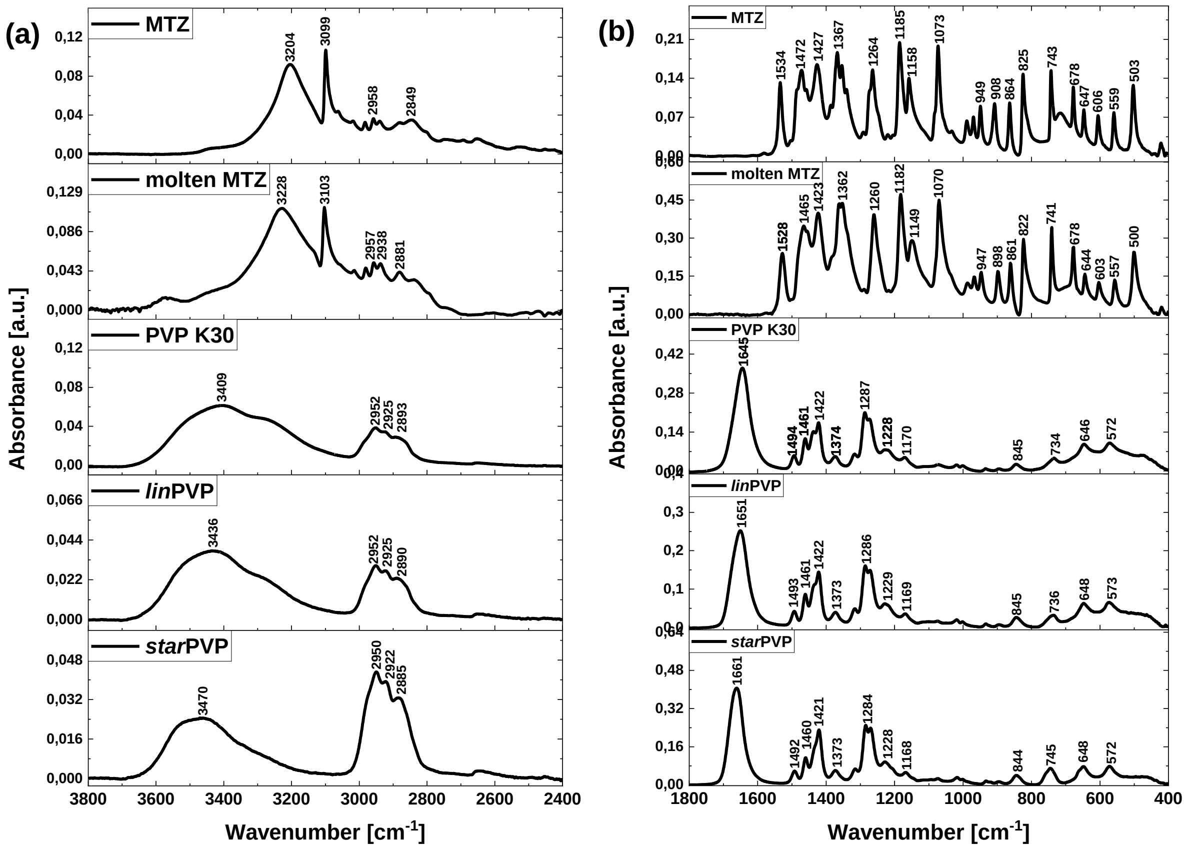

Figure S7. Infrared spectra of crystalline and molten MTZ as well as PVP samples. Data were presented in two spectral regions: (a) 3800–2400 cm^{-1} and (b)1800–400 cm^{-1}.

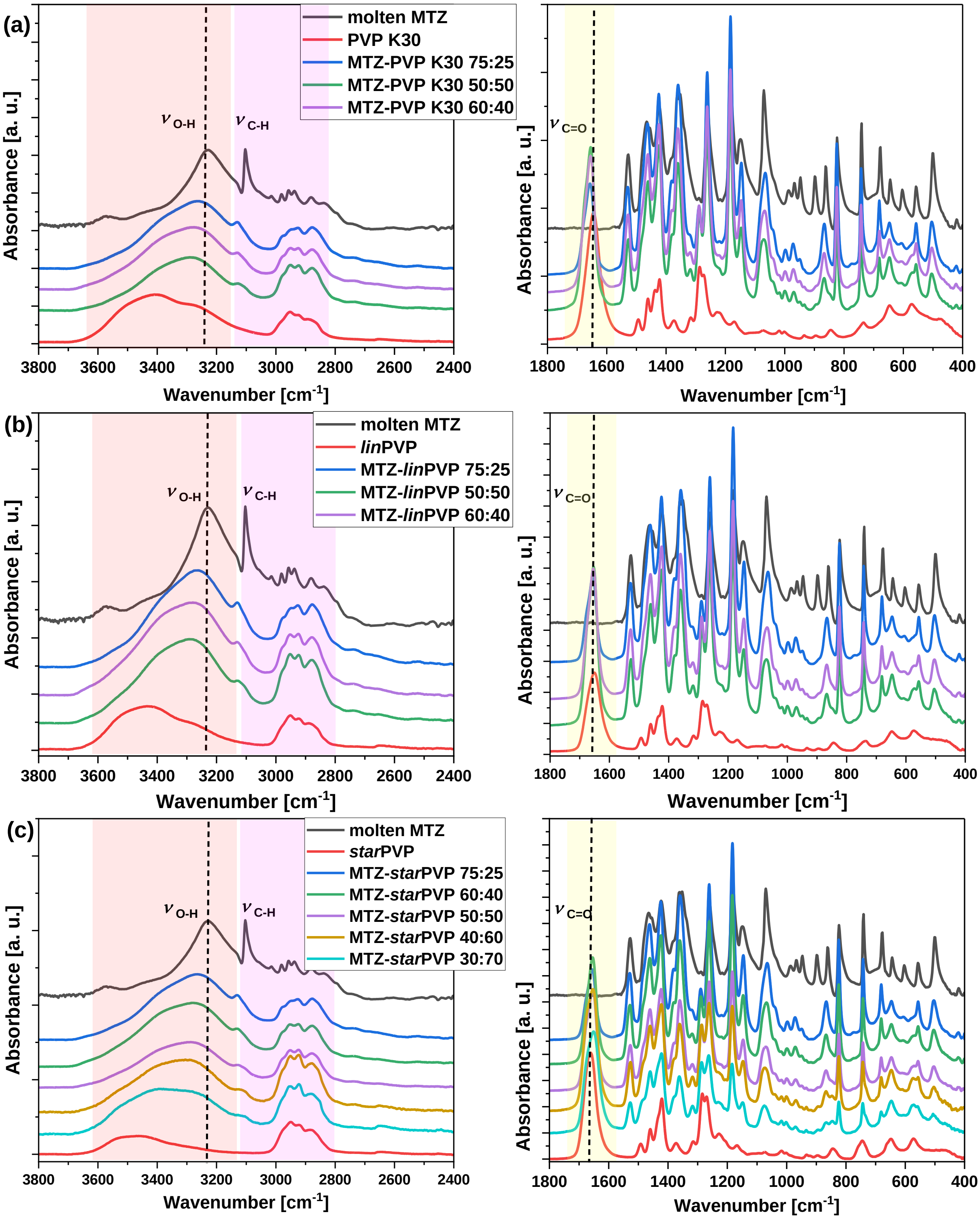

Figure S8. Infrared spectra of (a) MTZ-PVP K30, (b) MTZ-*lin*PVP, and (c) MTZ-*star*PVP binary mixtures prepared in different weight ratios, measured at T=295 K (in the supercooled liquid/or glassy states) together with the spectra registered for the molten MTZ and neat PVP polymers, presented in the ranges of 3800–2400 cm^{-1} (left) and 1800–400 cm^{-1} (right).

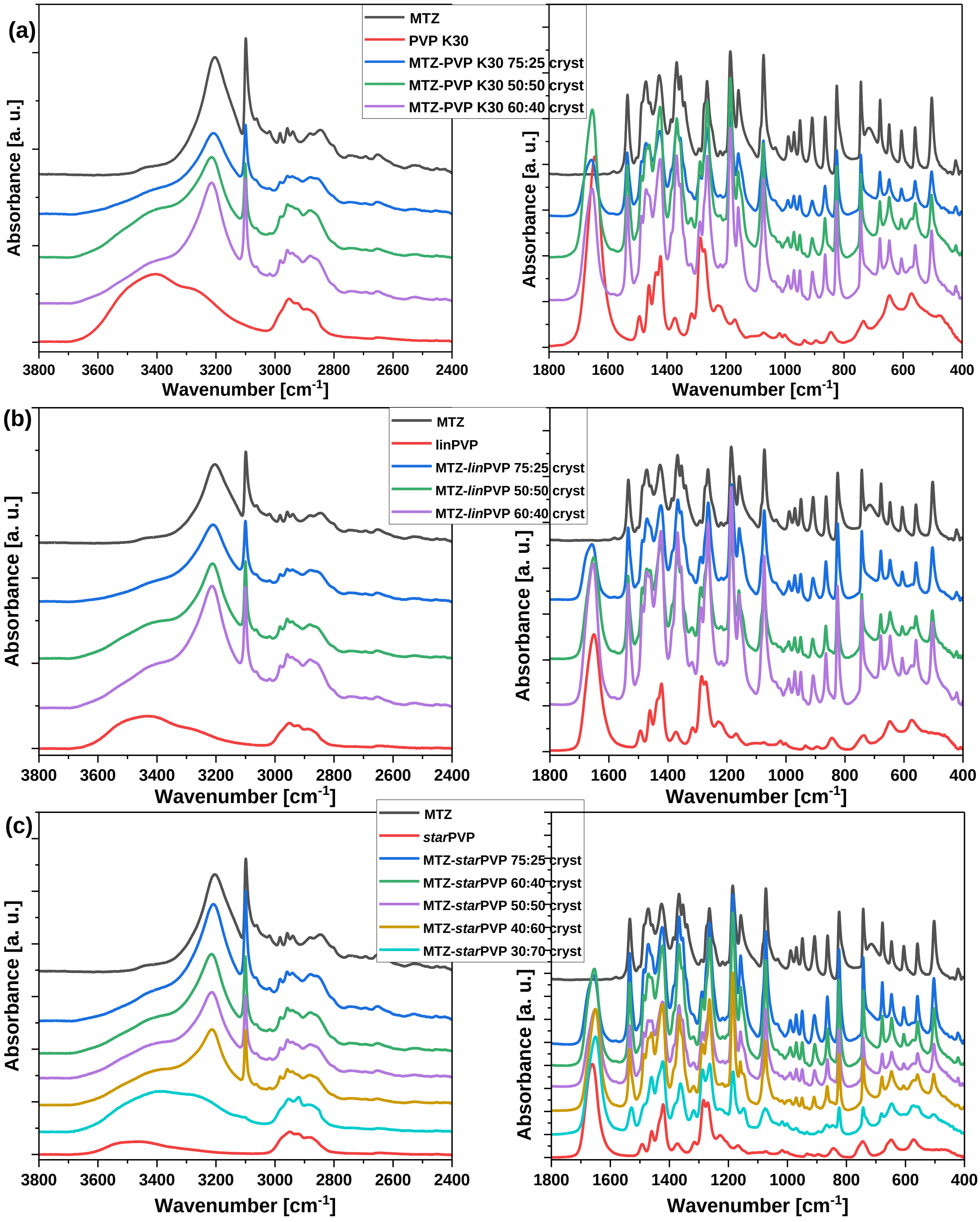

Figure S9. Infrared spectra of (a) MTZ-PVP K30, (b) MTZ-*lin*PVP, and (c) MTZ-*star*PVP binary mixtures (samples after recrystallization) prepared in different molar ratios together with the spectra registered for the crystalline MTZ and neat PVP polymers, presented in the ranges of 3800–2400 cm^{-1} (left) and 1800–400 cm^{-1} (right).

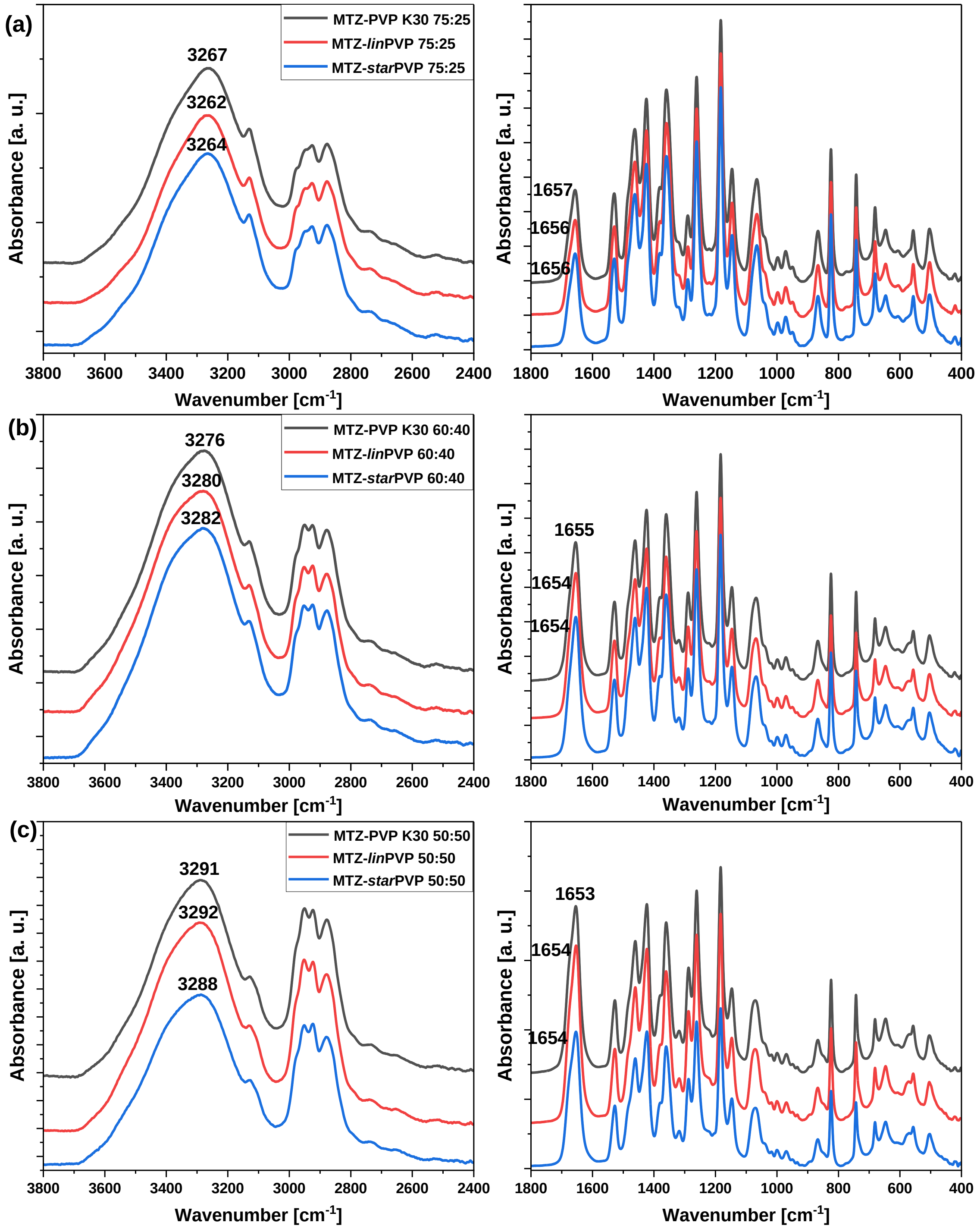

Figure S10. Infrared spectra of binary mixtures of MTZ with all PVP samples (K30, *lin*PVP and *star*PVP) ((a) 75:25, (b) 60:40, (c) 50:50 w/w) measured in the supercooled liquid state, presented in the ranges of 3800–2400 cm^{-1} (left) and 1800–400 cm^{-1} (right).

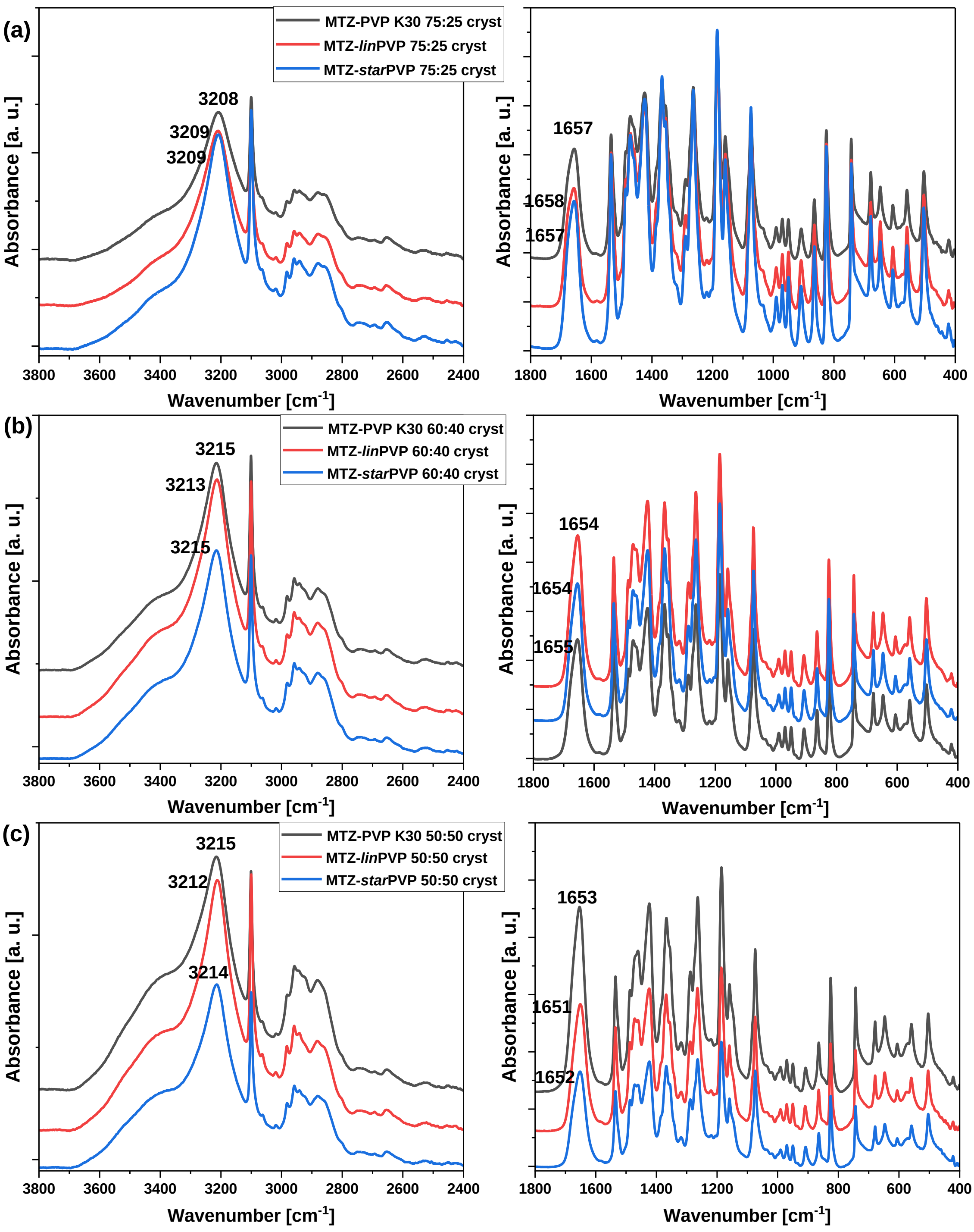

Figure S11. Infrared spectra of binary mixtures of MTZ with all PVP samples (K30, *lin*PVP and *star*PVP) ((a) 75:25, (b) 60:40, (c) 50:50 w/w) measured in the crystalline state, presented in the ranges of 3800–2400 cm^{-1} (left) and 1800–400 cm^{-1} (right).

3. DSC data

Table S1. The calorimetric glass transition temperature values (heating rate, ϕ =10 K/min) for neat PVPs and examined MTZ-PVP binary mixtures in various weight ratios.

Binary mixture, w/w	**Glass transition temperature, T_g [K]**		
	MTZ-PVP K30	MTZ-*lin*PVP	MTZ-*star*PVP
75:25	261.3	260.3	258.7
60:40	269.1	267.9	268.5
50:50	277.1	278.5	280.4
40:60	-	-	315.1
30:70	-	-	339.4
neat PVP	395.6	415.0	433.4

Table S2. The calorimetric melting temperature values (ϕ =10 K/min) for neat MTZ and the examined MTZ-PVP binary mixtures in various weight ratios.

Binary mixture, w/w	**Melting temperature, T_m [K]**		
	MTZ-PVP K30	MTZ-*lin*PVP	MTZ-*star*PVP
neat MTZ	437.3		
75:25	430.3	430.8	431.3
60:40	422.0	423.2	423.4
50:50	417.0	418.3	416.4

Table S3. The crystallization temperature values for MTZ-PVP K30 mixtures depending on the heating rate (ϕ).

**MTZ-PVP K30**			
ϕ [K/min]	T_c [K]		
	75:25	60:40	50:50
15	341.7		
12.5	336.7	389.7	
10	333.6	385.8	386.5
7.5	331.7	376.6	383.2
5	326.9	372.3	376.1
2.5			369.1

Table S4. The crystallization temperature values for MTZ-*lin*PVP mixtures depending on the heating rate (ϕ).

MTZ-*lin*PVP			
ϕ [K/min]	T_c [K]		
	75:25	60:40	50:50
15	335.1		
12.5	333.3	397.3	
10	330.3	388.5	387.7
7.5	327.1	382.6	385.2
5	322.5	359.1	381.8
2.5			375.8

Table S5. The crystallization temperature values for MTZ-*star*PVP mixtures depending on the heating rate (ϕ).

MTZ-*star*PVP			
ϕ [K/min]	T_c [K]		
	75:25	60:40	50:50
15	323.6		
12.5	321.2	394.5	
10	319.3	395.7	386.5
7.5	315.6	387.6	385.1
5	311.6	354.5	374.3
2.5			364.3

4. References:

1. Minecka, A.; Tarnacka, M.; Jurkiewicz, K.; Hachuła, B.; Wrzalik, R.; Bródka, A.; Kamiński, K.; Kamińska, E. The Impact of the Size of Acetylated Cyclodextrin on the Stability of Amorphous Metronidazole. *Int. J. Pharm.* **2022**, *624*, 122025, doi:10.1016/J.IJPHARM.2022.122025.
2. Bensouiki, S.; Belaib, F.; Sindt, M.; Rup-Jacques, S.; Magri, P.; Ikhlef, A.; Meniai, A.H. Synthesis of Cyclodextrins-Metronidazole Inclusion Complexes and Incorporation of Metronidazole - 2-Hydroxypropyl-β-Cyclodextrin Inclusion Complex in Chitosan Nanoparticles. *J. Mol. Struct.* **2022**, *1247*, 131298, doi:10.1016/j.molstruc.2021.131298.
3. Socrates, G. Infrared and Raman Characteristic Group Frequencies : Tables and Charts.; 2001.
4. Rahma, A.; Munir, M.M.; Khairurrijal; Prasetyo, A.; Suendo, V.; Rachmawati, H. Intermolecular Interactions and the Release Pattern of Electrospun Curcumin-Polyvinyl(Pyrrolidone) Fiber. *Biol. Pharm. Bull.* **2016**, *39*, 163–173, doi:10.1248/bpb.b15-00391.
5. Abdelghany, A.M.; Meikhail, M.S.; Oraby, A.H.; Aboelwafa, M.A. Experimental and DFT Studies on the Structural and Optical Properties of Chitosan/Polyvinyl Pyrrolidone/ZnS Nanocomposites. *Polym. Bull.* **2023**, *80*, 13279–13298, doi:10.1007/s00289-023-04700-0.
